# Moracin E and M isolated from *Morus alba* Linné induced the skeletal muscle cell proliferation via PI3K-Akt-mTOR signaling pathway

**DOI:** 10.1038/s41598-023-47411-2

**Published:** 2023-11-23

**Authors:** Hee Jae Kwak, Jinyoung Kim, Seo‐Young Kim, SeonJu Park, Junjeong Choi, Seung Hyun Kim

**Affiliations:** 1 Yonsei Institute of Pharmaceutical Sciences, College of Pharmacy, Yonsei University, Incheon, 21983 South Korea; 2 Division of Practical Application, Honam National Institute of Biological Resources, Mokpo, 58762 South Korea; 3 Chuncheon Center, Korea Basic Science Institute (KBSI), Chuncheon, 24341 South Korea

**Keywords:** Musculoskeletal abnormalities, Network topology

## Abstract

Twigs of *Morus alba* have been used in traditional medicine to treat muscle-related symptoms such as aches, numbness, and stiffness. Despite its clinical use in traditional medicine, its active compounds and mode of action have not yet been investigated. Therefore, we aimed to isolate the compounds from the twigs of *M. alba* and deduce active compounds, key gene targets, and mechanism of action against sarcopenia using network pharmacology analysis. Using various isolation techniques and spectroscopic methods, 43 phytochemicals, including 3 new flavonoids, were isolated and performed network pharmacology analysis. According to the computational-assistant analysis, 28 compounds, 9 genes, and the PI3K-Akt-mTOR signaling pathway were deduced as expected active compounds (EAC), key targets, and the main signaling pathway. To verify the predicted results, the cell proliferation activities of the EAC were evaluated. Especially, moracin E and M significantly increased by 130% (*p* < 0.001) and 57% (*p* < 0.05), respectively, which have more than 2- and 1.5-fold stronger effects compared to the control. Furthermore, both increased the expression level of proteins involved in the PI3K-Akt-mTOR signaling pathway and myogenic proteins, including myogenin and MyoD. This study demonstrated that moracin E and M exhibit cell proliferative effects on skeletal muscle cells through the PI3K-Akt-mTOR signaling pathway.

## Introduction

Sarcopenia is an age-related disorder characterized by progressive loss of muscle mass, strength, and function^1^. It has been recognized as a disease by an International Classification of Diseases-10 code in 2016^2^. Since then, the disease code has been assigned in many countries, including Germany and Australia^3,4^. Sarcopenia increases the risk of falls, and fractures, consequently, leading to functional dependence and disability^5,6^. All these changes reduce the quality of life of the elderly and increase the mortality rate. However, there is no treatment for sarcopenia officially approved by regulatory agencies such as the FDA and EMA. Therefore, research on the development of a treatment for sarcopenia is not only very important but also urgent.

Recently, herbal medicines and dietary supplements derived from natural products have been attracting attention as a way to prevent sarcopenia caused by various factors. *Morus alba* Linné is an important herbal resource, and its root barks, twigs, leaves, and fruits have been used as traditional medicines and dietary supplements to treat various diseases and symptoms^7,8^. Especially, twigs of *M. alba* have been used to treat aches, numbness in joints, and muscle stiffness in traditional Korean medicine. Moreover, its major components, prenylated flavonoids, coumarins, moracins, and stilbenes showed various pharmacological activities such as anti-inflammatory and anti-aging effects, suggesting the possibility that they may be active against sarcopenia^8–10^. Although natural products, such as *M. alba*, are an important source of therapeutic opportunities, their use may be limited due to technical barriers to getting detailed information for the use^11^. Identifying their active constituents is one of the most important steps in overcoming these limitations. However, the chemical complexity and diversity of natural products make investigating of active compounds very challenging. The recently emerged network pharmacology suggests an efficient approach to these difficulties^12,13^.

The present study applied network pharmacology to identify the muscle-related gene targets and signaling pathways involved in the action mechanism of *M. alba*. Network pharmacology has the advantages of overcoming the limitations of research on natural products by effectively deducing active compounds and their mode of action. Our previous studies have confirmed that this method is sufficiently effective and efficient for natural product research^14–16^. In this study, we isolated compounds from the twigs of *M. alba* and applied network pharmacology analysis to deduce the active compounds, key targets, and pharmacological mechanisms against sarcopenia. We further demonstrated in vitro effects of *M. alba* on cell proliferation of C2C12 skeletal muscle cells and validated its mechanism experimentally by western blot analysis. Our results suggest a novel therapeutic approach and perspective for treatment of sarcopenia using natural products.

## Results

### Structure elucidation of isolated compounds

**Figure 1:**
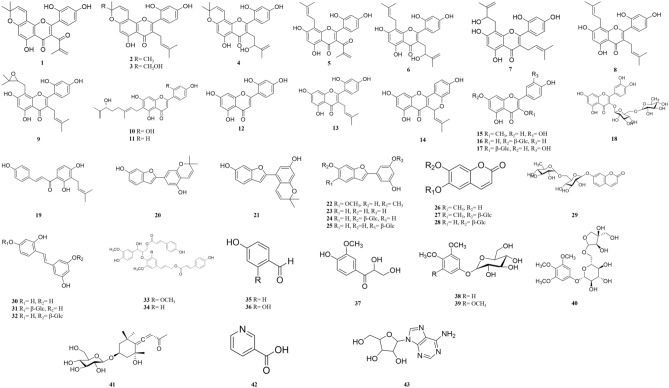
Isolated compounds from twigs of *M. alba.*

By means of various chromatographic resins and isolation techniques, three new flavonoids (**1**, **5** and **10**) along with forty known compounds were isolated (Fig. 1). All the isolates were elucidated by extensive spectroscopic methods including 1D and 2D NMR and HR‑ESI‑MS analysis.

Compound **1** was obtained as yellow amorphous powder and its molecular formula was determined as C_24_H_20_O_7_ by HR‐ESI‐MS ([M+HCOO])^−^-[H_2_O] ion at *m/z* 447.1082 (calcd for 447.1080). The ^1^H NMR spectrum (Fig. S2, Table S1) showed typical ABX‐type aromatic ring signals at *δ* 7.04 (1H, d, *J* = 8.2 Hz, H‐6′), 6.31 (1H, d, *J* = 2.8 Hz, H‐3′) and 6.30 (1H, dd, *J* = 2.8, 8.2 Hz, H‐5′) and a singlet signal at *δ* 6.10 (1H, s, H‐6). In addition, the spectrum displayed two singlets at *δ* 5.73 (1H, s, H‐3″a) and 5.96 (1H, s, H‐3″b), a coupled doublets at *δ* 5.54 (1H, d, *J* = 10.2 Hz, H‐2‴) and 6.51 (1H, d, *J* = 10.1 Hz, H‐1‴), and three methyl groups at *δ* 1.37 (6H, s, H‐4‴ and ‐5‴) and *δ* 1.76 (3H, s, H‐4″). These proton signals together structure with the ^13^C NMR (Fig. S2, Table S1) signals corresponded to a backbone of 7,8‐pyranoflavone with a methacryloyl group. In addition, the HMBC correlation (Figs. S3 and 8) of H‐4″ to C‐1″, C‐2″ and C‐3″, as well as from H‐3″ to C‐1″, C‐2″ and C‐4″, supported the presence of the methacryloyl moiety, and its connectivity was determined at C‐3 by the reference of morustralin B^17^. Furthermore, The HMBC correlations from H‐4‴ to C‐3‴ and C‐5‴, from H‐1‴ to C‐7, C‐8, C‐8a and C‐3‴, as well as from H‐6 to C‐4a, C‐5, C‐7 and C‐8, determined the pyrano ring to be fused at C‐7 and C‐8. The ABX signal aromatic ring was confirmed by the HMBC correlation of H‐3′ to C‐1′ and C‐5′, H‐5′ to C‐1′ and C‐3′, and H‐6′ to C‐1′, respectively (Figs. S3 and 8). From all the above evidence, the planar structure of compound **1** was established as 2‐(2,4‐dihydroxyphenyl)‐5‐hydroxy‐3‐methacryloyl‐8,8‐dimethyl‐4*H*,8*H*‐pyrano[2,3‐*f*]chromen‐4‐one and named as morusalbalin A.

Compound **5** was isolated as yellow amorphous powder, C_24_H_22_O_7_ as the molecular formula was determined by HR‐ESI‐MS spectrum for ([M+HCOO]^−^)-[H_2_O] ion at *m/z* 449.1214 (calcd for 449.1236). The ^1^H NMR (Fig. S4, Table S1) showed the presence of an ABX group at *δ* 7.01 (1H, d, *J* = 8.3 Hz, H‐6′), 6.29 (1H, dd, *J* = 2.3, 8.3 Hz H‐5′) and 6.31 (1H, d, *J* = 2.2 Hz, H‐3′) and one singlet at *δ* 6.17 (1H, s, H‐6). The ^13^C NMR (Fig. S4, Table S1) showed a carbonyl resonance at *δ* 183.5 and six aromatic carbon signal at *δ* 164.5, 163.0, 162.3, 160.7, 158.0, 157.3, which were similar to the NMR spectral data of mornigrol E (**6**)^18^. The HMBC spectrum (Figs. S5 and 8) showed the correlations of H‐6 to C‐4a/C‐5/C‐7/C‐8; H‐3′ to C‐1′/C‐2′/C‐4′/C‐5′; H‐5′ to C‐1′/C‐3′; H‐6′ to C‐2/C‐2′/C‐4′; H‐4″ to C‐1″/C‐2″/ C‐3″ and H‐3″ to C‐1″/C‐2″/C‐4″. According to the above data and comparing to the data of **6** and **1** suggest that **5** was 5,7,2′,4′‐four hydroxyl flavonoid with 1‐keto‐2‐methylallyl (methacryloyl) substituent. Moreover, The ^1^H NMR spectrums of *δ* 3.28 (2H, d, *J* = 7.3 Hz, H‐1‴), 5.10 (1H, t, *J* = 7.3 Hz, H‐2‴), 1.53 (3H, s, H‐5‴), 1.50 (3H, s, H‐4‴) assured that the presence of isopentene group. In the HMBC experiment, H‐1‴ showed correlations with C‐7/C‐8/C‐8a/C‐2‴; H‐2‴ with C‐1‴/C‐4‴/C‐5‴. These results suggested that the isopentene group located at C‐8 in ring A (Figs. S5 and 8). Thus, the structure of **5** was confirmed as 2‐(2,4‐dihydroxyphenyl)‐5,7‐dihydroxy‐3‐methacryloyl‐8‐(3‐methylbut‐2‐en‐1‐yl)‐4*H*‐chromen‐4‐one and named as morusalbalin B.

Compound **10** was a yellow amorphous powder. The molecular formula of **10** was determined to be C_25_H_26_O_7_ by HR‐ESI‐MS spectrum for [M−H]^−^ ion at *m/z* 437.1606 (calcd for 437.1600). The ^1^H NMR (Fig. S6, Table S1) spectrum exhibited signals of ABX aromatic system proton at *δ* 7.67 (1H, d, *J* = 8.8 Hz, H‐6′), 6.36 (1H, dd, *J* = 2.3, 8.8 Hz, H‐5′) and 6.34 (1H, d, *J* = 2.3 Hz, H‐2′); two singlets at *δ* 6.50 (1H, s, H‐3) and 6.37 (1H, s, H‐8); and a 7″‐hydroxy‐3″,8″‐dimethylocta‐2″,8″‐dienyl group at *δ* 5.20 (1H, t, *J* = 7.2 Hz, H‐2″), 4.76 (1H, overlap, H‐9″a), 4.69 (1H, t, *J* = 1.8, H‐9″b), 3.87 (1H, t, *J* = 6.6, H‐7″), 3.27 (2H, m, H-1″), 1.90 and 1.52 (each 2H, m, H‐5″, 6″), 1.72 (3H, s, H‐4″), and 1.60 (3H, s, H‐10″). The ^13^C NMR spectrum showed 25 carbon signals, including two methyls, four methylenes, seven methines, and twelve quaternary carbons (Fig. S6, Table S1). The above data indicated that **10** was similar to (7″R)‐(−)‐6‐(7″‐hydroxy‐3″,8″‐dimethyl‐2″,8″‐octadien‐1″‐yl)apigenin (**11**)^10^. The HMBC spectrum (Figs. S7 and 8) showed H-1″ correlated with C-5 (*δ* 159.9), C-6 (*δ* 112.9) and C-7 (*δ* 163.6) that confirmed the 7″‐hydroxy‐3″,8″‐dimethylocta‐2″,8″‐dienyl group located at C-6 in ring A. Therefore, the structure of compound **10** was established as 2-(2,4-dihydroxyphenyl)-5,7-dihydroxy-6-(6-hydroxy-3,7-dimethylocta-2,7-dien-1-yl)-4*H*-chromen-4-one and named as morusalbalin C. Additionally, known compounds were identified by comparing with the spectral data of the previously reported studies (Result S1).

### Network pharmacology analysis

#### Drug-likeness (DL) and oral bioavailability (OB) evaluation of isolated compounds

All the isolates were evaluated for DL and OB using the QED method to select the EAC (Table S2). The parameters required to evaluate the QED and OB were obtained using the method as in previous studies^14–16^. The cut-off values of QED and OB for the selection of EAC were set to 0.3 or more and TRUE, respectively. Based on these cut-off values, 28 compounds were selected as EAC (Table S3).

#### Acquisition of potential targets

**Figure 2:**
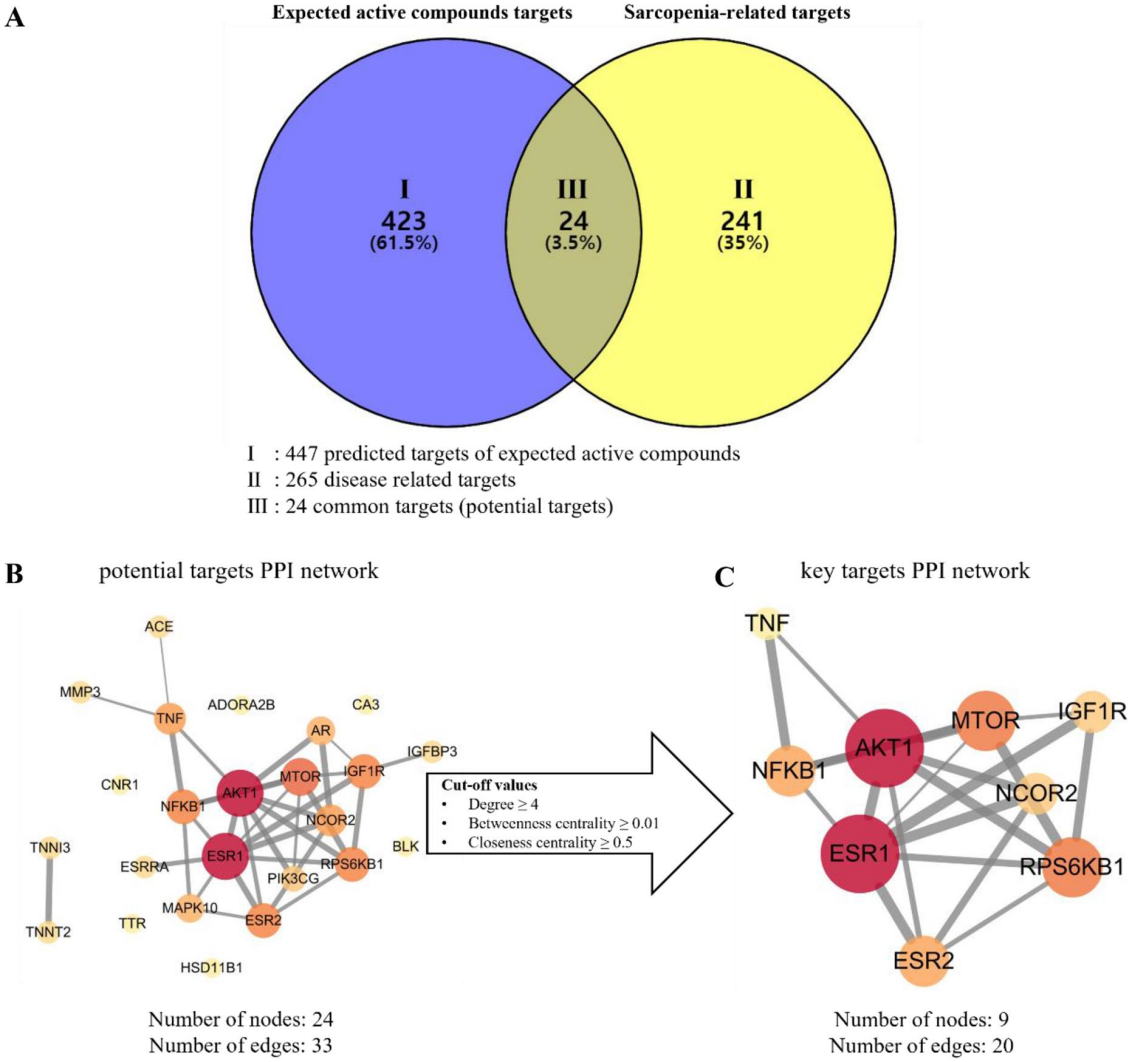
Acquisition of potential targets and analysis of PPI networks. (**A**) A venn diagram of expected active compounds predicted targets and sarcopenia-related targets. (**B**) PPI network of potential targets. (**C**) PPI network of key targets.

The SwissTargetPredction database was used to aquire targets of the EAC and any duplicated or false-positive targets were removed. As a result, 447 targets were predicted in total. Moreover, 265 sarcopenia-related targets were obtained from the GeneCards database. As shown in Fig. 2A, 24 target genes that were commonly included in both sets were selected as the potential targets (Table S4).

#### Construction and analysis of the PPI networks of potential and key targets

**Figure 3:**
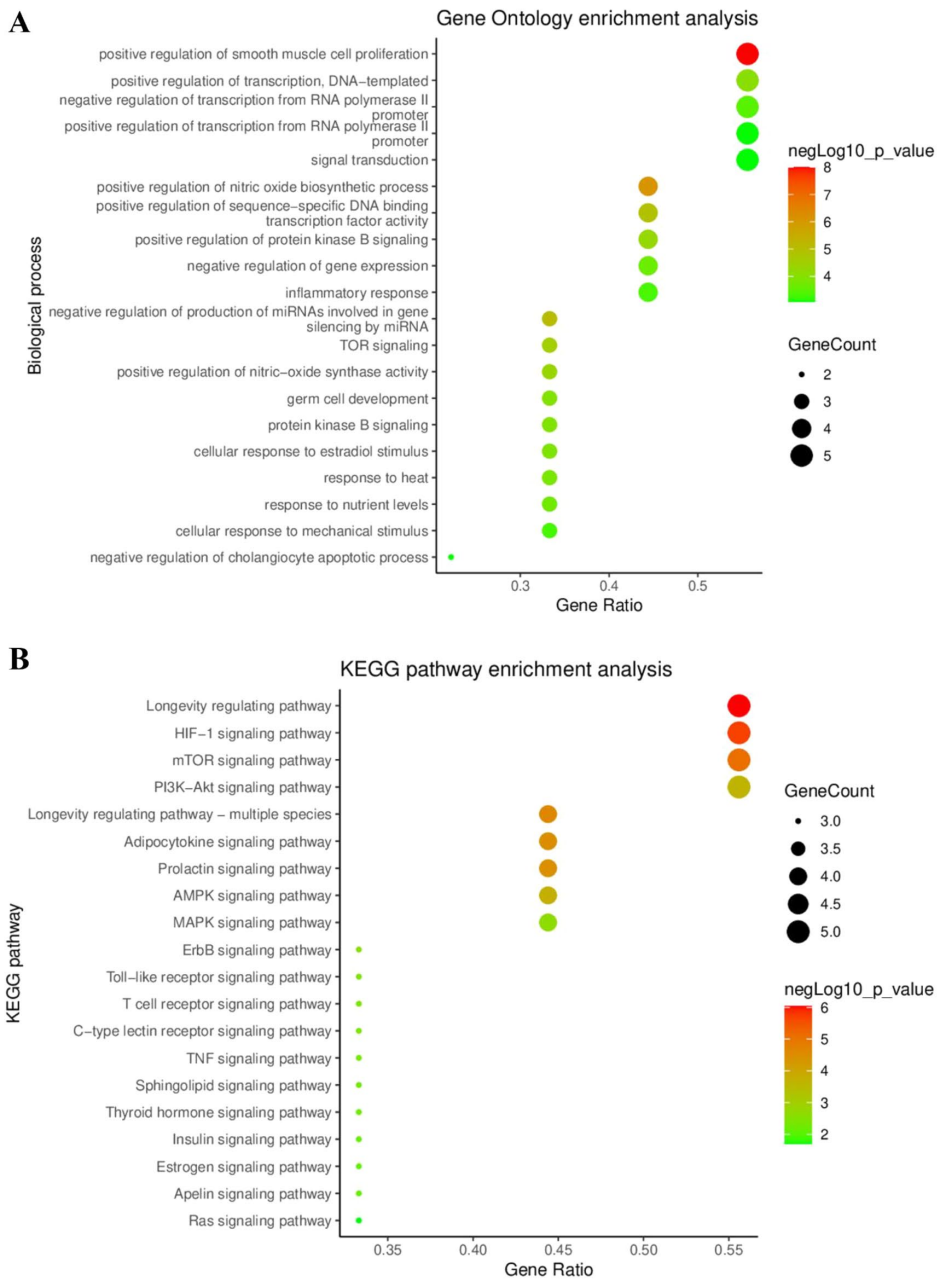
Bubble charts of GO and KEGG pathway enrichment analysis. (**A**) Bubble char of GO enrichment analysis: top 20 biological process. (**B**) Bubble chart of top 20 KEGG pathways.

Construction of network was done as described previously^14–16^. The PPI network of the 24 potential targets consisted of 24 nodes and 33 edges (Fig. 2B). To identify the key targets, cut-off values were set as follows: degree ≥ 4, betweenness centrality ≥ 0.01, and closeness centrality ≥ 0.5. Among the targets, 9 fulfilled requirements  (TableS5). Moreover, the PPI network of these key targets consisted of 9 nodes and 20 edges (Fig. 3C). Among the key targets, IGF1R (insulin like growth factor 1 receptor), AKT1 (AKT serine/threonine kinase 1), RPS6KB1 (ribosomal protein S6 kinase B1), and MTOR (mechanistic target of rapamycin kinase) showed significant values in topological network analysis.

#### Gene Ontology (GO) and Kyoto Encyclopedia Genes and Genomes (KEGG) pathway enrichment analysis of key targets

The DAVID 2021 database was used for GO and KEGG pathway enrichment analyses to predicted the biological processes and signaling pathways associated with the key targets. From the GO analysis, a total of 78 biological process terms were obtained, and we selected 20 based on their *p*-values. As shown in Fig. 3A, key targets were tightly associated with regulation of smooth muscle cell proliferation, nitric oxide biosynthesis, and other biological processes. According to the results from the KEGG pathway analysis, 66 KEGG terms were acquired, and 20 terms with highest *p*-value were selected. The key target genes were found to be mainly concentrated in the Longevity regulating, HIF-1, PI3K-Akt, and mTOR signaling pathway (Fig. 3B). Specifically, the longevity regulating pathway showed the highest *p*-value and gene ratio and it included multiple signaling pathways such as PI3K-Akt-mTOR, FOXO, AMPK, and NF-κB signaling pathways. These are associated with regulating protein synthesis, autophagy, apoptosis, inflammatory, and oxidative metabolism.

#### Construction and analysis of integrated network

**Figure 4:**
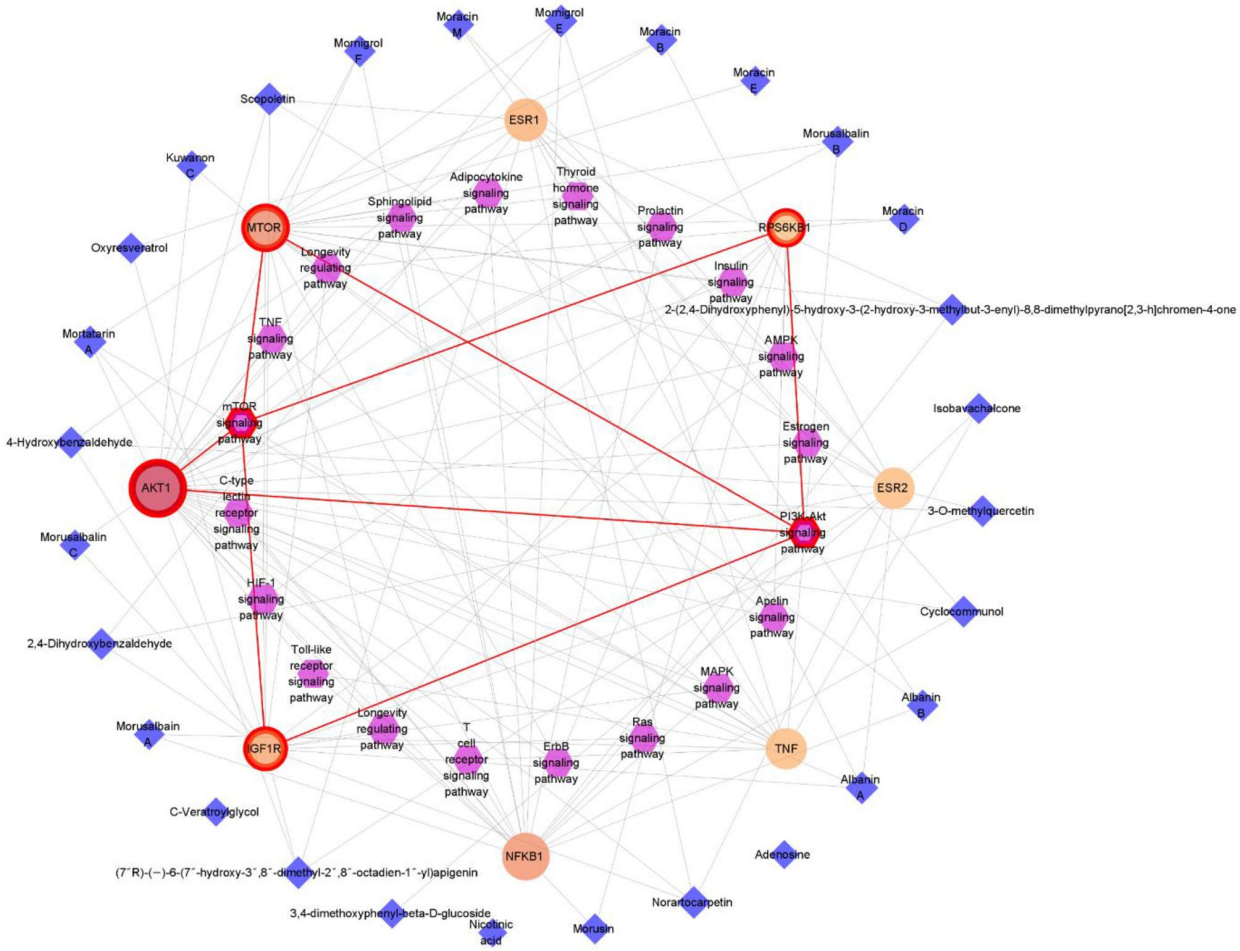
Integrated Expected active compounds-Key targets-Pathways network.

The three categories, EAC, key targets, and signaling pathways, were merged to construct an EAC-key targets-pathways (C-T-P) network (Fig. 4). By analyzing the C-T-P network, it is possible to obtain comprehensive information about the initial individual analyses and to reveal the complex relationships that govern pharmacological mechanisms. According to topological analysis, PI3K-Akt, mTOR and Longevity signaling pathways showed the highest degree values of 5 in the pathway nodes. These pathways exhibited a high gene ratio and *p*-values in KEGG enrichment analysis. Additionally, AKT and mTOR showed degree values of 32 and 28, respectively. These protein genes were involved in the 20 pathways with a high frequency, which indicates that they may play a crucial role in the enrichment pathway. Moreover, these genes have been shown as important targets in the PPI network analysis.

### Experimental validation

#### Cell proliferation activity of EAC

**Figure 5:**
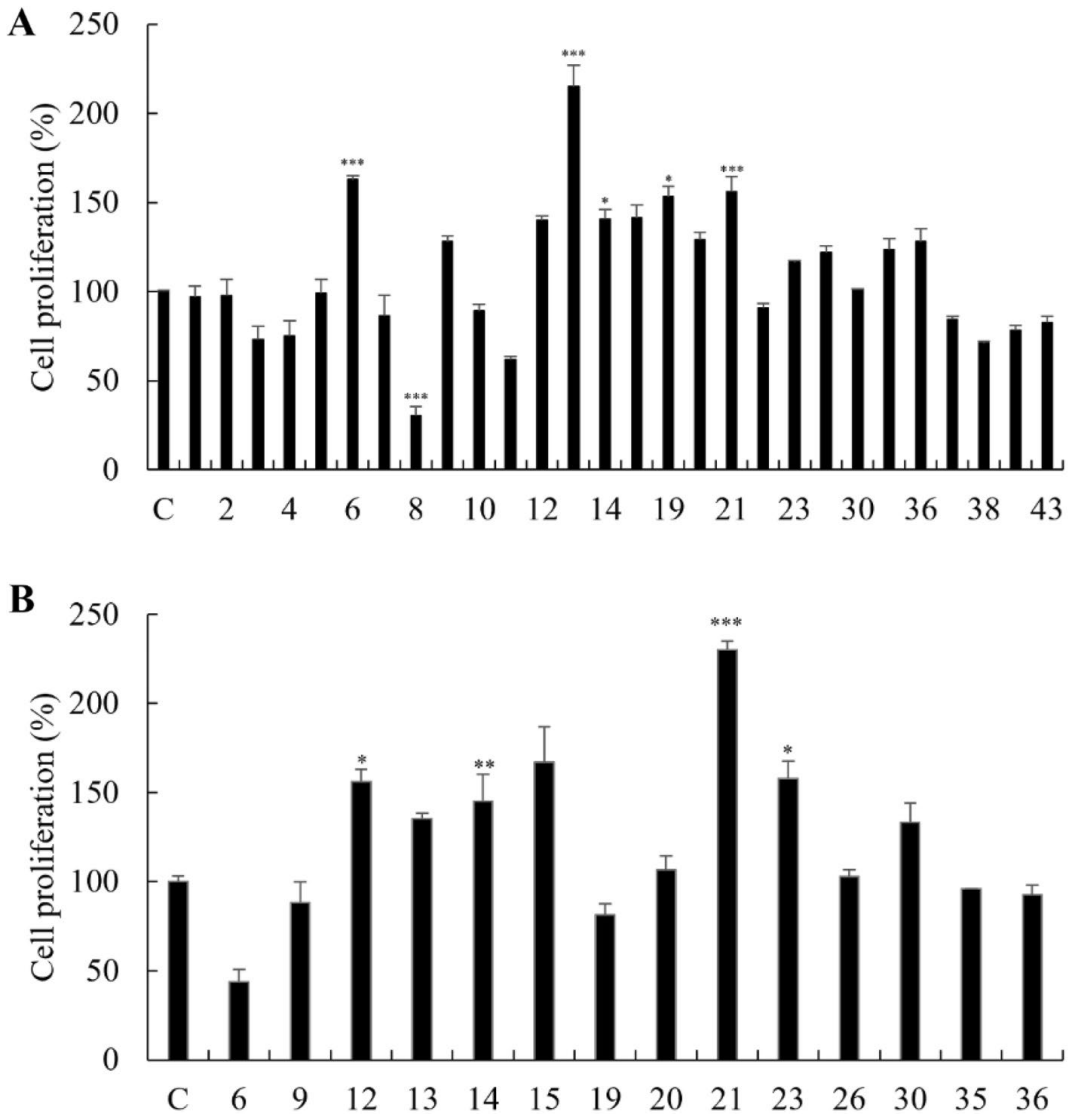
Cell proliferation activity of the EAC. (**A**) Cell proliferation activity of the 28 EAC on C2C12 cells by MTT assay. (**B**) Cell proliferation activity of 14 active compounds on C2C12 cell by BrdU assay. C: control. Experiments were performed in triplicate and the data were expressed as mean ± SEM; *p < 0.05, **p < 0.01, ***p < 0.001 as compared to the control (untreated cells).

Prior to evaluating the cell proliferation activities of EAC, their cytotoxicity was measured by the 3‐(4‐5‐dimethylthiazoly‐2‐yl)‐2,5‐diphenyl‐2H‐tetrazolium bromide (MTT) assay. All the tested compounds did not exhibit a cytotoxic effects on C2C12 cells at the concentration of 25 μM (Fig. S1). The EAC were assessed for their skeletal muscle cell proliferation activity in C2C12 cells at 25 μM using MTT assay. As shown in Fig. 5A, 14 compounds (**6**, **9**, **12**‐**15**, **19**‐**23**, **26**, **30**, **35** and **36**) were significantly increased proliferation of myoblasts compared to non‐treated cells. However, the others did not affect cell proliferation, and several compounds (**3**, **4**, **7**, **8**, **10**, **11**, **22**, **37**‐**42**) remarkably reduced cell proliferation. These results may be caused by cell toxicity due to three treatments of the compounds during the cell differentiation process. The cell proliferative effects of 14 compounds were evaluated using the 5‐bromo‐2′‐deoxyuridine (BrdU) assay to confirm their definitive skeletal muscle cell proliferation activity. All the tested compounds were evaluated at the final concentration of 25 μM. Among the tested compounds, compounds **12**‐**15**, **21**, **23** and **30** significantly increased proliferation of myoblasts (Fig. 5B). However, some compounds (**6**, **9** and **19**) showed cell toxicity during the cell differentiation period. Among the proliferative compounds, moracin E (**21)** showed the highest cell proliferation activity and moracin M (**23)** which belongs to the same chemical classification as **21** also exhibited remarkable efficacy on cell proliferation. Therefore, we selected these moracin derivatives further evaluate their effects on skeletal muscle cell growth. Furthermore, we conducted an assessment of cell toxicity for Moracin E (**21**) and Moracin M (**23**) across a concentration range of 0, 0.3, 1, 3, 10, 30, and 50 μM, exposing cells for 4 h, 24 h, and 48 h (Fig. S14). Moracin E (**21**) demonstrated negligible cell toxicity, with a maximum reduction in cell viability to 83%, surpassing the 75% threshold. In contrast, Moracin M (**23**) demonstrated slight cell toxicity at a concentration of 50 μM, reducing cell viability to 73%. However, concentrations below 30 μM showed no significant impact on cell viability. All compounds were dissolved in Dimethyl sulfoxide (D8418, Sigma-Aldrich), and the control samples were treated solely with Dimethyl sulfoxide.

#### Regulation of skeletal muscle cell growth by moracin E and M via PI3K-Akt-mTOR signaling pathway

**Figure 6:**
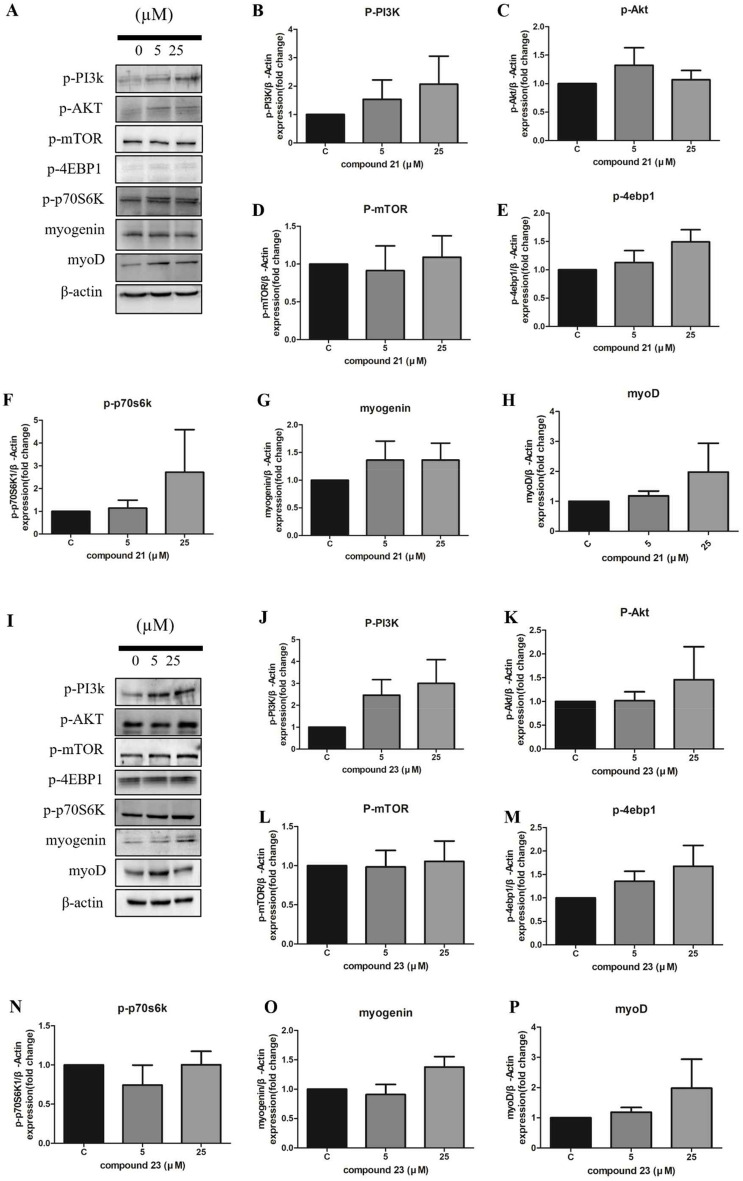
Mouse skeletal muscle cell differentiation and proliferation effect of moracin E and M. Evaluation of muscle cell differentiation by measuring myogenic protein expression. Evaluation of muscle cell proliferation by measuring phosphorylated proteins of PI3K/AKT/mTOR pathway. (**A**) 5 µM and 25 µM of moracin E were treated with differentiation culture media during the differentiation of C2C12. (**B**–**H**) Each protein level was determined using image J(software) following normalized by β-Actin level. (**I**) 5 µM and 25 µM of moracin M were treated during the differentiation of C2C12. (**J**–**P**) Each protein level was determined using image J(software) following normalized by β-Actin level. (n = 3; representative of 3 biological replicates per group). Original blots/gels are presented in Supplementary Figs. 12 and 13.

As these compounds previously showed a high correlation with PI3K-AKT-mTOR signaling on integrated network analysis, we identified whether these exactly work in vitro cell culture systems. Throughout the differentiation period, mouse myoblast C2C12 cells (CRL-1772, ATCC) were treated separately with concentrations of 5 μM and 25 μM for both moracin E (**21**) and moracin M (**23**). When C2C12 cells were cultured with moracin E (**21**) and moracin M (**23**) separately, there was an observed increase in the levels of p-PI3K (phosphorylated PI3K), p-AKT, and p-p70S6K proteins, as well as myogenic transcriptional factors MyoD and Myogenin, when compared to non-treated cells (Fig. 6). Interestingly, both compounds did not alter p-mTOR levels, while p-4EBP1 was unexpectedly elevated when compared to non-treated cells. . It is worth noting that the phosphorylation of mTOR typically activates p70S6K, a key player in muscle protein synthesis, while concurrently inhibiting 4EBP1, which is involved in cap-binding complex-mediated translation and ribosomal biogenesis^19^. Contrary to our expectations, our results indicate that moracin treatment did not lead to the activation of mTOR, and although p-4EBP1 increased, 4EBP1 was not inhibited. Despite this, p70S6K activation was observed, promoting muscle protein synthesis. In conjunction with PI3K-AKT pathway activation, there was an increase in Myogenin and MyoD levels compared to non-treated cells.

## Discussion

Recently, natural products have been attracting attention as a novel approach to alleviate the progression and symptoms of sarcopenia^20^. Traditional medicines in most countries, including Korea, have used natural products, especially plant-derived ones, as medicine. In many cases, their active constituents and mechanisms of action have not been clarified compared to their clinical uses and efficacy. In other words, it is likely that the study of natural products used in traditional medicines will yield the discovery of new active compounds or mechanisms of action that are efficacious for the treatment of some diseases such as sarcopenia. In this study, twigs of *M. alba* were selected for this purpose, since it has been used for muscle-related disease in traditional Korean medicine.

In order to more clearly understand the effects and mechanisms of action of *M. alba*, phytochemical isolation and identification were conducted first, and as a result, the 43 chemical components were isolated from its twigs. Then we utilized network pharmacology analysis to efficiently deduce active compounds among them, inferring 28 compounds (**1**‐**15**, **19**‐**23**, **26**, **30**, **35**‐**38**, **42** and **43**) as EAC against sarcopenia. Most of them linked to the target genes such as AKT1, RPS6KB1, MTOR and IGF1R which showed correlation with PI3K-Akt-mTOR signaling pathway in the C-T-P network (Fig. 4). Relationship between the four targets and the signaling pathway have been demonstrated in several studies. Akt stimulates activation of mTOR, resulting in phosphorylation of p70S6K (RPS6KB1), which promotes protein synthesis by activating ribosomal protein S6^21^. In addition, IGF1R is activated by the binding of IGF-1, which then induces phosphorylation of the PI3K-Akt pathway, resulting in differentiation and survival of myoblasts^22^. Taken together, these evidences suggest that the components, targets and pathway predicted through network pharmacology analysis are sufficiently reasonable inferences.

**Figure 7:**
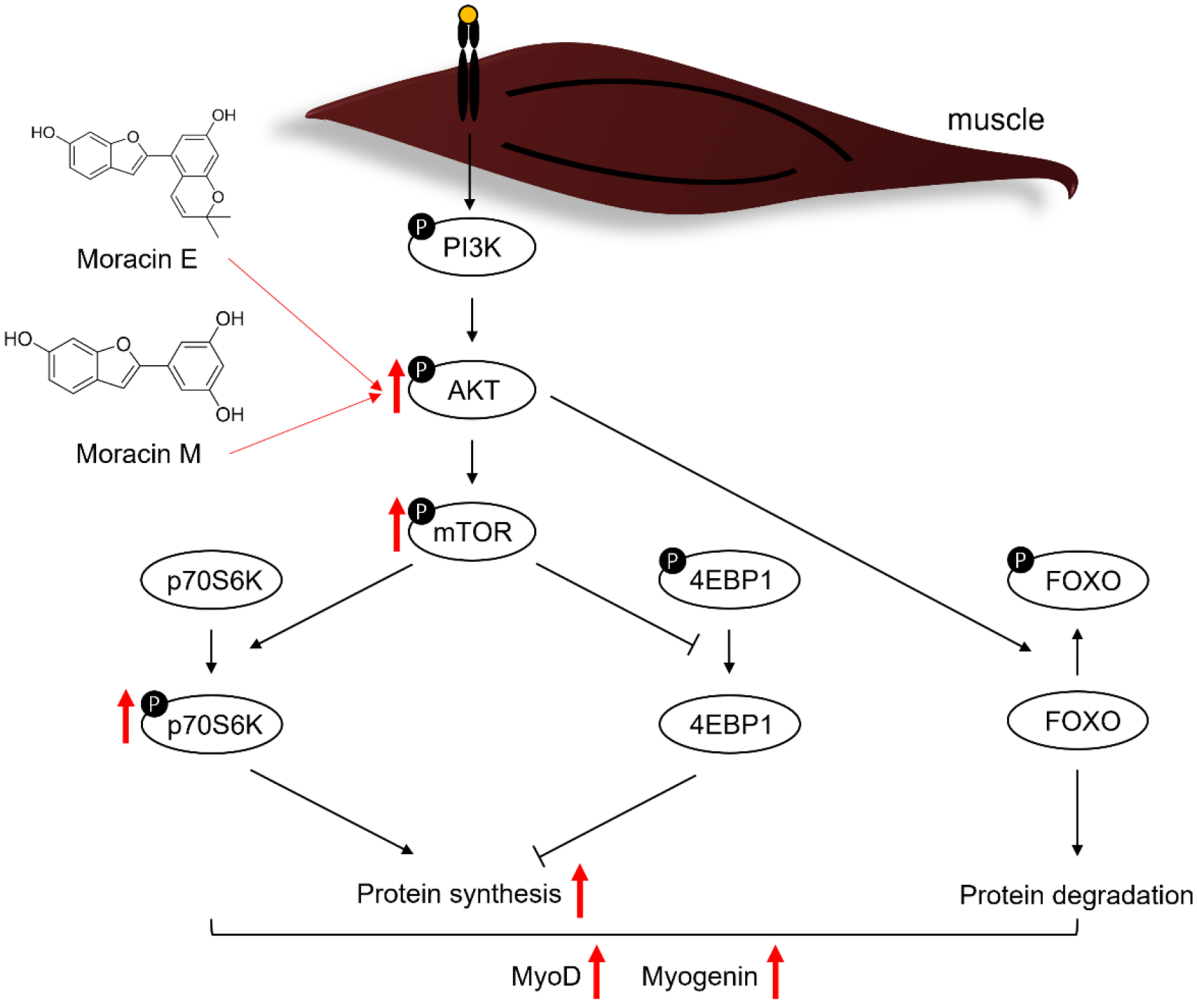
Schematic illustration of the mechanism of moracin E and M.

These results were experimentally validated. Among EAC, 7 compounds (**12**‐**15**, **21**, **23** and **30**) showed muscle cell proliferative activities in both MTT and BrdU assays. Four are classified as flavonoids (**12**‐**15**), two as moracins (**21**, **23**) and one as a stilbene (**30**). Although previous research has demonstrated the molecular mechanisms of flavonoids and stilbenes on skeletal muscle health^23,24^, that of moracins has not been explored. Therefore, we focused on investigating the effects of moracins on skeletal muscle cell growth and the underlying mechanisms. To confirm whether our candidate compounds promote myogenic differentiation, we compared the expression level of each protein involved in the PI3K-Akt-mTOR signaling pathway and myogenic proteins using western blotting. Myogenin also known as MyoG is crucial in the early stages of myogenesis and myofiber development^25^ and MyoD is a myogenic regulator that occurs mainly during myocyte fusion^26^. These two important components of muscle health were both increased in cells treated with moracins (Fig. 6). Moracin E and M activated PI3K-Akt-mTOR signaling pathway, a crucial pathway for myoblast differentiation and survival. While mTOR activation was not found to be significant, thereby not leading to p-4ebp1 inhibition through dephosphorylation, these compounds were observed to increase the active form of AKT and sustain the phosphorylation of p70S6K, consequently enhancing muscle protein synthesis. Furthermore, even though the expression of p-mTOR did not increase substantially, the persistent phosphorylation of p70S6K indicated enhanced ribosomal protein synthesis, coinciding with elevated levels of myogenic proteins. In summary, our findings collectively suggest that Moracin E and M promote myogenic differentiation in myoblasts (Fig. 7).

## Conclusion

In this study, we investigated the active compounds from the twigs of *M. alba*, a natural product used in traditional medicine, and predicted their sarcopenia associated targets via data-mining based approach, the network pharmacology analysis. Experimental validations were conducted to verify the results inferred from the network pharmacology analysis and revealed that two moracins, moracin E and M, promoted the growth of skeletal muscle cells by triggering the phosphorylation of Akt, mTOR, and p70S6K in PI3K-Akt-mTOR signaling pathway. In conclusion, our study not only identifies novel natural product-based therapeutics for sarcopenia, but also suggests an efficient method for discovering new active compounds from traditional medicinal herbs and interpreting their mechanism of action.

## Methods

### Plant material

The dried twigs of *M. alba* were purchased from Humanherb Co., Ltd. (Lot No. S4619071, Daegu, Korea) in July 2020. This sample was cultivated in Yeongcheon, Gyeongsangbuk-do, Korea. A voucher specimen (TMA-202007) was deposited at the Herbarium of College of Pharmacy, Yonsei Institute of Pharmaceutical Sciences, Yonsei University, Incheon, Korea. The collection and purchasing of the plant material and related research complies with relevant institutional, national and international guidelines and legislation.

### Investigation of chemical components

The dried twigs of *M. alba* (18.0 kg) were extracted with 100% MeOH (5 L × 3 times) under sonication for 4 h at room temperature. After filtration and evaporation under reduced pressure, the MeOH extract (73.70 g) was suspended in water and successively partitioned with n‐hexane, chloroform (CHCl_3_) and ethyl acetate (EtOAc) to obtain CHCl_3_‐, EtOAc‐, and water‐soluble fractions, respectively. Compounds isolation was performed using diverse isolation techniques including, open column chromatography and prep-HPLC under various conditions (Experimental S2).

### Network pharmacology analysis

The network pharmacology was performed according to the methods described in our previously reported studies^14–16^. Briefly, SwissADME database^27^ was used to obtain physicochemical properties of the isolates. Then, drug-likeness and oral bioavailability were estimated by the Quantitative Estimate of Drug-likeness (QED)^28^ and Veber’s rule^29^ using obtained the physicochemical properties, and we selected expected active compounds (EAC). SwissTargetPrediction and GeneCards databases^30,31^ were applied to collect targets of expected active compounds and disease-related, and common targets were selected as potential targets. STRING database^32^ was used to get protein–protein interaction (PPI) of the potential targets. The potential targets PPI network was constructed and analyzed using Cytoscape (3.9.1) to choose key targets. Biological processes and signaling pathways involved in the key targets were analyzed and visualized using DAVID 2021 database^33^ and ImageGP^34^. Thereafter, an integrated network, expected active compounds-key targets-pathways network, was established and analyzed using Cytoscape.

### Cell culture and differentiation

Mouse myoblast cell line C2C12 are (purchased from ATCC, CRL-1772) grown in Dulbecco’s Modified Eagle Medium (DMEM) supplemented with 10% heat-inactivated Fetal Bovine Serum (FBS) and 1% penicillin‐streptomycin for 48 h until the confluency reached 70%. Culture media was changed to differentiation media containing DMEM with 2% Horse Serum (HS) and 1% antibiotics for 6 days with medium changes every other day.

### Skeletal muscle cell proliferation activity

Cell proliferation effect of the EAC on C2C12 skeletal muscle cells was measured by MTT assay according to the previous reported studies^35–37^. Throughout the differentiation period, once every two days, the medium was substituted with a differentiation medium (DMEM containing 2% HS, and 1% streptomycin‐penicillin) and the EAC were treated. Subsequently, cell proliferation was measured at the 540 nm absorbance; cell proliferation activity of the EAC‐treated cells was calculated and compared to 100% of non‐treated cells.

A more reliable cell proliferation activity was evaluated by a 5‐bromo‐2′‐deoxyuridine (BrdU) assay for compounds exhibiting proliferative activity in the MTT assay. The BrdU cell proliferation assay is a non‐isotopic assay for the in‐vitro quantitative detection of newly synthesized DNA of actively proliferating cells. Briefly, after 48 h of the initial cell seeding with 5 × 104 cells per well (96 well plate), the growth medium (DMEM containing 10% FBS) was replaced with a differentiation medium (DMEM containing 2% HS) and treated compounds every other day during differentiation period. Finally, 6 days after the differentiation induction, cell proliferation activity was calculated by comparison with the absorbance at 450 nm of the standard solution of BrdU in the non‐treated cells.

### Protein sampling and western blot analysis

After differentiation, cells were washed with cold phosphate‐buffered saline buffer. Following, the cells are lysed with Radioimmunoprecipitation assay (RIPA) buffer (gendepot, R4100‐010) containing a duo cocktail (gendepot, p3300) for protein isolation. Total proteins were separated by sodium dodecyl sulphated‐polyacrylamide gel electrophoresis (SDS‐PAGE) and transferred to nitrocellulose membrane. The membranes were obstructed using 5% bovine serum albumin in TBS‐T (Tris‐buffered saline with 0.1% Tween 20) and incubated overnight at 4℃ with individual primary antibody. Following wash procedure with TBS‐T, it was incubated in secondary antibodies which are conjugated with horse‐radish peroxidase for 1 h at room temperature. Protein bands were visualized using a chemiluminescent substrate (ECL Western, Thermo Scientific, 32106) and captured using FusionCapt Advance software. Antibodies used for western blot were as follows: p-PI3K p85(1:1000, SC423; Santa Cruz), p-AKT(1:500, 4060S; Cell Signaling Technology), p-mTOR(1:1000, 5536S; Cell Signaling Technology), p-p70S6K(1:1000, SC8416; Santa Cruz), p-4E-BP1(1:1000, 9459S; Cell Signaling Technology), MyoD(1:1000, SC377460; Santa Cruz), Myogenin(1:1000, SC12732;Santa Cruz), β-Actin(1:1000, SC47778; Santa Cruz), Goat anti-mouse IgG secondary antibody HRP conjugated(1:2000, 62-6520; Invitrogen), Goat anti-rabbit IgG secondary antibody HRP conjugated(1:2000, 31460; Invitrogen). For quantification, protein levels were normalized by β-Actin bands and analyzed using Image J software.

### Statistical analysis

Statistical significance was assessed through one‐way analysis of variance (ANOVA) and multiple comparisons with Bonferroni correction. Statistical significance was set at p < 0.05. All statistical analyses were carried out utilizing GraphPad Prism software (version 5).

### Ethics statement

This research does not involve the ethics of human and animal experiments.

### Relevant institutional, national, and international guidelines and legislation

The authors confirm that all methods were performed in accordance with the relevant guidelines in the “Methods” section.

### Supplementary Information

Supplementary Information.

## Data Availability

All data generated or analyzed during this study are included in this published article (and its Supplementary data file).
