# Supplementary material for: Moracin E and M isolated from Morus alba Linné induced the skeletal muscle cell proliferation via PI3K-Akt-mTOR signaling pathway
Source: Sci Rep. 2023 Nov 23;13:20570. doi: 10.1038/s41598-023-47411-2 (PMC10667267; doi:10.1038/s41598-023-47411-2)
Supplement: Supplementary file 1 — Supplementary Information. [file 41598_2023_47411_MOESM1_ESM.docx]

**Supplementary data**

**Moracin E and M isolated from *Morus alba* Linné induced the skeletal muscle cell proliferation via PI3K**-**Akt**-**mTOR signaling pathway**

**Authors**

Hee Jae Kwak^1,†^, Jinyoung Kim^1,†^, Seo‐Young Kim^2^, SeonJu Park^3^, Junjung Choi^1^, Seung Hyun Kim^1,*^

**Affiliations**

^1^Yonsei Institute of Pharmaceutical Sciences, College of Pharmacy, Yonsei University, Incheon 21983, Korea

^2^Division of practical application, Honam National Institute of Biological Resources, Mokpo, 58, 762, Korea

^3^Chuncheon Center, Korea Basic Science Institute (KBSI), Chuncheon 24341, Korea

^†^These authors contributed equally to this work.

***Corresponding Author**

Seung Hyun Kim

Tel.: +82-32-749-4514; fax: +82-32-749-4105

E-mail address: [kimsh11@yonsei.ac.kr](mailto:kimsh11@yonsei.ac.kr)**Result S1. Structure elucidation of known compounds.**

The known compounds, morusin (**2**) [1], albanin B (**3**) [2],2‐(2,4‐dihydroxyphenyl)‐5‐hydroxy‐3‐(2‐hydroxy‐3‐methylbut‐3‐enyl)‐8,8‐dimethylpyrano[2,3‐*h*]chromen‐4‐one (**4**) [3], mornigrol E, F (**6**‐**7**) [4], kuwanon C (**8**) [5], mortatarin A (**9**) [6], (7″R)‐(−)‐6‐(7″‐hydroxy‐3″,8″‐dimethyl‐2″,8″‐octadien‐1″‐yl)apigenin (**11**) [7], norartocarpetin (**12**) [8], albanin A (**13**) [9], cyclocommunol (**14**) [10], 3‑*O*‑methylquercetin (**15**) [11], kaempferol 7‐*O*‐*β*‐D‐glucoside (**16)** [12], quercetin 3‐*O*‐*β*‐D‐glucoside (**17**) [13], rutin (**18**) [14], isobavachalcone (**19**) [15], moracin D (**20**) [16], moracin E (**21**) [17], moracin B (**22**) [18], moracin M (**23**) [19], moracin M 6‐*O*‐*β*‐D‐glucoside, moracin M 3′‐*O*‐*β*‐D‐glucoside (**24‐25**) [20], scopoletin (**26**) [21], scopolin (**27**) [22], cichoriin (**28**) [23], umbelliferone‐7‐*O*‐*α*‐rhamnopyranosyl‐(1‐6)‐*β*‐D‐glucoside (**29**) [24], oxyresveratrol (**30**) [25], oxyresveratrol 4‐*O*‐*β*‐D‐glucooside (**31**) [26], oxyresveratrol 3′‐*O*‐*β*‐D‐glucoside (**32**) [27], dadahol A, B (**33**‐**34**) [28], 4‐hydroxybenzaldehyde (**35**) [29], 2,4‐dihydroxybenzaldehyde (**36**) [30], C‑veratroylglycol (**37**) [31], 3,4‐dimethoxyphenyl‐*β*‐D‐glycoside (**38**) [32], 3,4,5‐trimethoxyphenyl‐*β*‐D‐glycoside, kelampayoside A (**39**‐**40**) [33], icariside B_1_ (**41**) [34], nicotinic acid (**42**) [35], adenosine (**43**) [36] were identified by comparison of their NMR and MS data with those reported in the literature.

**Experimental S1. Materials**

Thin layer chromatography (TLC) was performed on silica gel 60 F254 (0.25 mm, Merck) and RP 18 F254S (0.25 mm, Merck) plates. For column chromatography (CC), silica gel (230–400 mesh, Merck, Billerica, MA, USA), YMC ODS resins (150 μm, Mitsubishi Chemical Ltd., Kasugai, Aichi, Japan) were used. Preparative HPLC was carried out using an AGILENT 1260 HPLC system using with Waters Atlantis dC18 OBD prep column (10 mm i.d. × 150 mm, 10μm) and YMC‐Pack Pro C18 RS (10 mm i.d. × 150 mm, 5μm). All NMR spectra were recorded on Bruker 600‐MR‐NMR and Agilent 400‐MR‐NMR spectrometer operated at 600/150 MHz and 400/100 MHz for 1H and 13C, respectively. Data processing was carried out with the MestReNova ver. 12.0.1 program. HR‐ESI‐MS data were obtained using an Agilent 6550 iFunnel Q-TOF system equipped with 1290 Infinity binary pump, 1290 Infinity auto-sampler, 1290 Infinity PDA detector. Data processing was carried out with Mass Hunter Data acquisition and Qualitative analysis software (Agilent).

The twigs of *M. alba* were purchased from Humanherb Co., Ltd. (Lot No. S4619071, Daegu, Korea) in July 2020. The sample was authenticated by Prof. Seung Hyun Kim of the Yonsei Institute of Pharmaceutical Sciences, Yonsei University, Korea. A voucher specimen (TMA‐202007) was deposited at the Herbarium of College of Pharmacy, Yonsei Institute of Pharmaceutical Sciences, Yonsei University, Incheon, Korea.

C2C12, a mouse myoblasts cell line, was purchased from the American Type Culture Collection (ATCC, Manassas, VA, USA). Dulbecco’s Modified Eagle Medium (DMEM), fetal bovine serum (FBS), horse serum (HS), penicillin-streptomycin, trypsin-EDTA, and Dulbecco’s Phosphate Buffered Saline (DPBS) were acquired from Gibco-BRL (Burlington, Ont, Canada). All the other chemicals used were of analytical grade.

**Experimental S2. Isolation of compounds from twigs of *M. alba***

The CHCl_3_ fraction (12.30 g) was subject to a silica gel column chromatography and eluting with a gradient of CHCl_3_:MeOH (20:1 → 10:1, v/v) yielding two fractions: MA2A and MA2B. The MA2A fraction was chromatographed on a silica gel column eluting with CHCl_3_:MeOH (15:1, v/v) yielding three fractions: MA3B, MA3C and MA3D. The MA3B fraction was chromatographed on a YMC RP‐18 column eluting with MeOH:water (7:1, v/v) yielding a fraction, MA3B1. The MA3B1 was subjected to HPLC system, eluted with 14% aq. MeCN to yield scopoletin (**26**, 3.9 mg). The MA3C fraction was chromatographed on a YMC RP‐18 column eluting with MeOH:water (5:1, v/v) yielding four sub-fractions (3C2: 46.9 mg, 3C3: 28.7 mg, 3C4: 25.9 mg, 3C5: 128.8 mg). The 3C2 to 3C5 fractions were chromatographed on HPLC using Atlantis dC18 OBD column with 42, 44, 47, and 61% aq. MeCN, respectively, yielding moracin B (**22**, 1.0 mg), moracin D (**20**, 2.0 mg), moracin E (**21**, 1.0 mg), cyclocommunol (**14**, 4.6 mg), morusalbalin A (**1**, 3.5 mg), isobavachalcone (**19**, 1.8 mg), morusin (**2**, 39.0 mg). Subsequently, MA3D fraction was chromatographed on a YMC RP‐18 column eluting with MeOH:water (7:1, v/v) yielding two sub‐fractions (3D1: 239.7 mg, 3D2: 145.0 mg). The 3D1 and 3D2 fractions were chromatographed on HPLC using Atlantis dC18 OBD column, eluting with 43 and 50% aq. MeCN to yield mornigrol E (**6**, 4.2 mg), morusalbalin B (**5**, 7.6 mg), mornigrol F (**7**, 16.8 mg), mortatarin A (**9**, 12.1 mg), 2‐(2,4‐dihydroxyphenyl)‐5‐hydroxy‐3‐(2‐hydroxy‐3‐methylbut‐3‐enyl)‐8,8‐dimethylpyrano[2,3‐*h*]chromen‐4‐one (**4**, 6.4 mg), kuwanon C (**8**, 112.1 mg). The MA2B fraction was chromatographed on a silica gel column eluting with CHCl_3_:MeOH (8:1, v/v) yielding a MA3F fraction, which was purified further on HPLC using the same condition except for the eluting solvent of 43% aq. MeCN to yield (7″R)‐(−)‐6‐ (7″‐hydroxy‐3″,8″‐dimethyl‐2″,8″‐octadien‐1″‐yl)apigenin (**11**, 3.7 mg).

The EtOAc fraction (13.06 g) was chromatographed on a silica gel column eluting with gradient of CHCl_3_:MeOH (10:1 → 2.5:1, v/v) yielding two fractions, MA4A and MA4C. The MA4A fraction was chromatographed on a YMC RP‐18 column eluting with MeOH:water (9:1, v/v) yielding two sub‐fractions: MA5B and MA5D. The MA5B fraction was chromatographed on a silica gel column eluting with MeOH:water (5:1, v/v) yielding two small fractions (5B1: 28.4 mg, 5B2: 54.7 mg). The 5B1 further chromatographed on HPLC using Atlantis dC18 OBD column with 30% aq. MeCN, yielding 4‐hydroxybenzaldehyde (**35**, 1.4 mg), 2,4‐dihydroxybenzaldehyde (**36**, 1.2 mg). The 5B2 fraction was eluted with the same HPLC conditions except for the solvent composition of 41% aq. MeCN to yield dadahol A (**33**, 6.6 mg) and dadahol B (**34**, 5.5 mg). The MA5D fraction was applied to a YMC RP‐18 column, and by eluting with MeOH:water (4:1, v/v) to yield three sub-fractions (5D1: 1.35 g, 5D2: 233.7 mg, 5D3: 67.5 mg). The 5D1 further chromatographed on YMC RP‐18 column eluting with MeOH:water (1.5: 1, v/v) yielding three small fractions (5D11: 140.2 mg, 5D12: 51.9 mg, 5D13: 107.8 mg). Subsequently, theses fractions also eluted with the HPLC using Atlantis dC18 OBD column with 20, 34 and 25% aq. MeCN, respectably, to yield oxyresvertrol (**30**, 73.8 mg), moracin M (**23**, 20.6 mg), norartocarpetin (**12**, 12.4 mg) and 3‑*O*‑methylquercetin (**15**, 4.9 mg). 5D2 and 5D3 fractions were eluted the same HPLC conditions except for the eluting solvent of 38 and 51% aq. MeCN to yield albanin A (**13**, 10.1 mg), morusalbanin C (**10**, 1.1 mg) and albanin B (**3**, 4.6 mg). The MA4C fraction was chromatographed on a silica gel column eluting with CHCl_3_:MeOH (1.25:1, v/v) yielding three fractions: MA5I, MA5J and MA5K. All of three fractions applied to a prep‐HPLC using Atlantis dC18 OBD column, 16, 20 and 25% aq. MeCN, to yield oxyresveratrol 4‐*O*‐*β*‐D‐glucooside (**31**, 3.8 mg), oxyresveratrol 3′‐*O*‐*β*‐D‐glucoside (**32**, 2.6 mg), moracin M 6‐*O*‐*β*‐D‐glucoside (**24**, 1.0 mg), moracin M 3′‐*O*‐*β*‐D‐glucoside (**25**, 6.7 mg), kaempferol 7‐*O*‐*β*‐D‐glucoside (**16**, 1.0 mg), and quercetin 3‐*O*‐*β*‐D‐glucoside (**17**, 4.3 mg).

The water fraction (41.24 g) was chromatographed on a Diaion HP‑20 column and eluted with 75 and 50% MeOH to yield two sub‑fractions, MA6B, MA6C. The MA6B fractions was subjected to a silica gel column, and by eluting with CHCl_3_:MeOH:water (3:1:0.15, v/v/v) to yield five partial‐fractions (6B2: 60.1 mg, 6B3: 157 mg, 6B4: 48.8 mg, 6B5: 75.0 mg, 6B6: 1.48 g). All of five sub‐fractions were chromatographed on HPLC using Atlantis dC18 OBD column, eluting with 10, 11, 13, 12, 19% aq. MeCN, to yield C‑veratroylglycol (**37**, 1.4 mg), icariside B1 (**41**, 1.8 mg), cichoriin (**28**, 1.9 mg), kelampayoside A (**40**, 2.6 mg), umbelliferone‐7‐*O*‐*α*‐rhamnopyranosyl‐(1‐6)‐*β*‐D‐glucoside (**29**, 1.8 mg), rutin (**18**, 1.9 mg). The MA6C fraction also applied to a same condition of silica gel column chromatography to yield two sub‑fractions (6C2: 51.4 mg, 6C3: 110.8 mg). Two small fractions were chromatographed on same HPLC system except eluting solvent concentration of 10, 8% aq. MeCN, yielding rutin (**42**, 2.6 mg), 3,4‐dimethoxyphenyl‐*β*‐D‐glycoside (**38**, 2.0 mg), 3,4,5‐trimethoxyphenyl‐*β*‐D‐glycoside (**39**, 7.5 mg), scopolin (**27**, 4.7 mg) and adenosine (**43**, 23.2 mg).

**Experimental S3. Measurement of cell viability**

C2C12 cell viability was analyzed using the MTT assay according to the method described in our previous reported studies [37-39]. After 24 h of cell seeding with 1 🞨 10^5^ cells per well (96 well plate), the 28 expected active compounds (25 μM) were dissolved in DMSO and treated for additional 24 h. After, MTT stock solution (50 μL, 2 mg/mL in DPBS) was added to each well for 4 h 37 ℃. The formazan crystals were dissolved in DMSO, and the absorbance was measured using an ELISA plate reader at 540 nm (BioTek Instruments, Inc., Winooski, VT, USA). The survival rate of the 28 expected active compounds‐treated cells was calculated and compared to 100% of non‐treated cells.


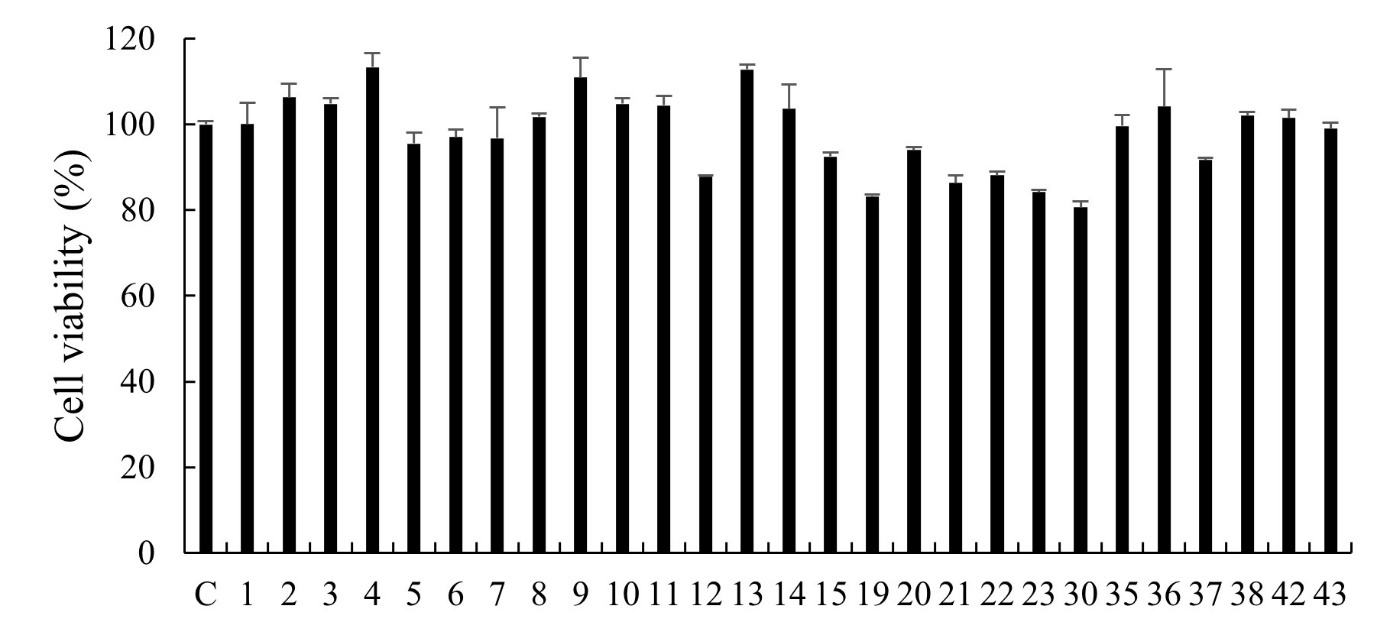


**Figure S1. Cytotoxicity of the 28 expected active compounds on C2C12 cells.**

C: control. Experiments were performed in triplicate and the data were expressed as mean ± SEM.

**
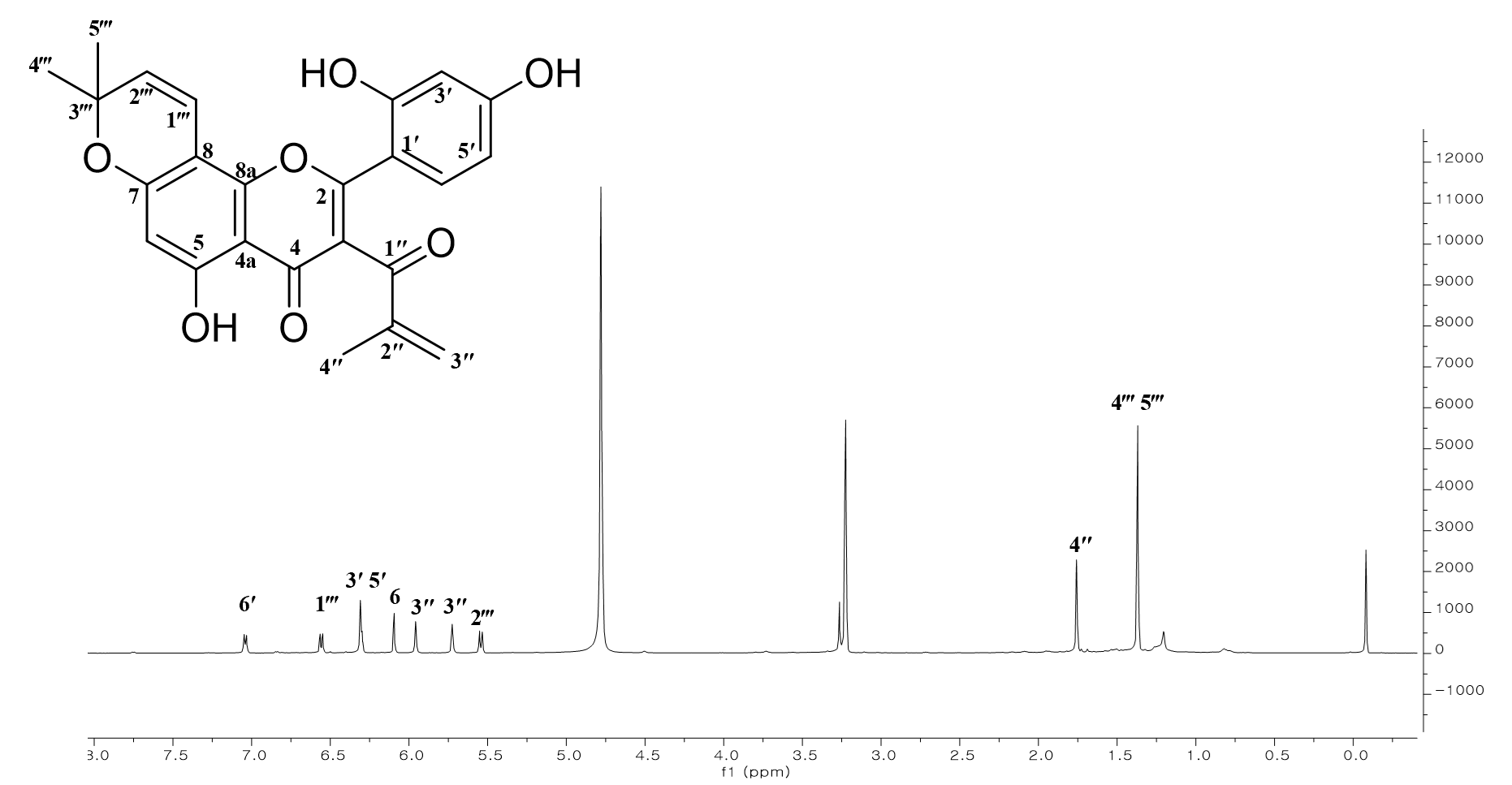
**

**
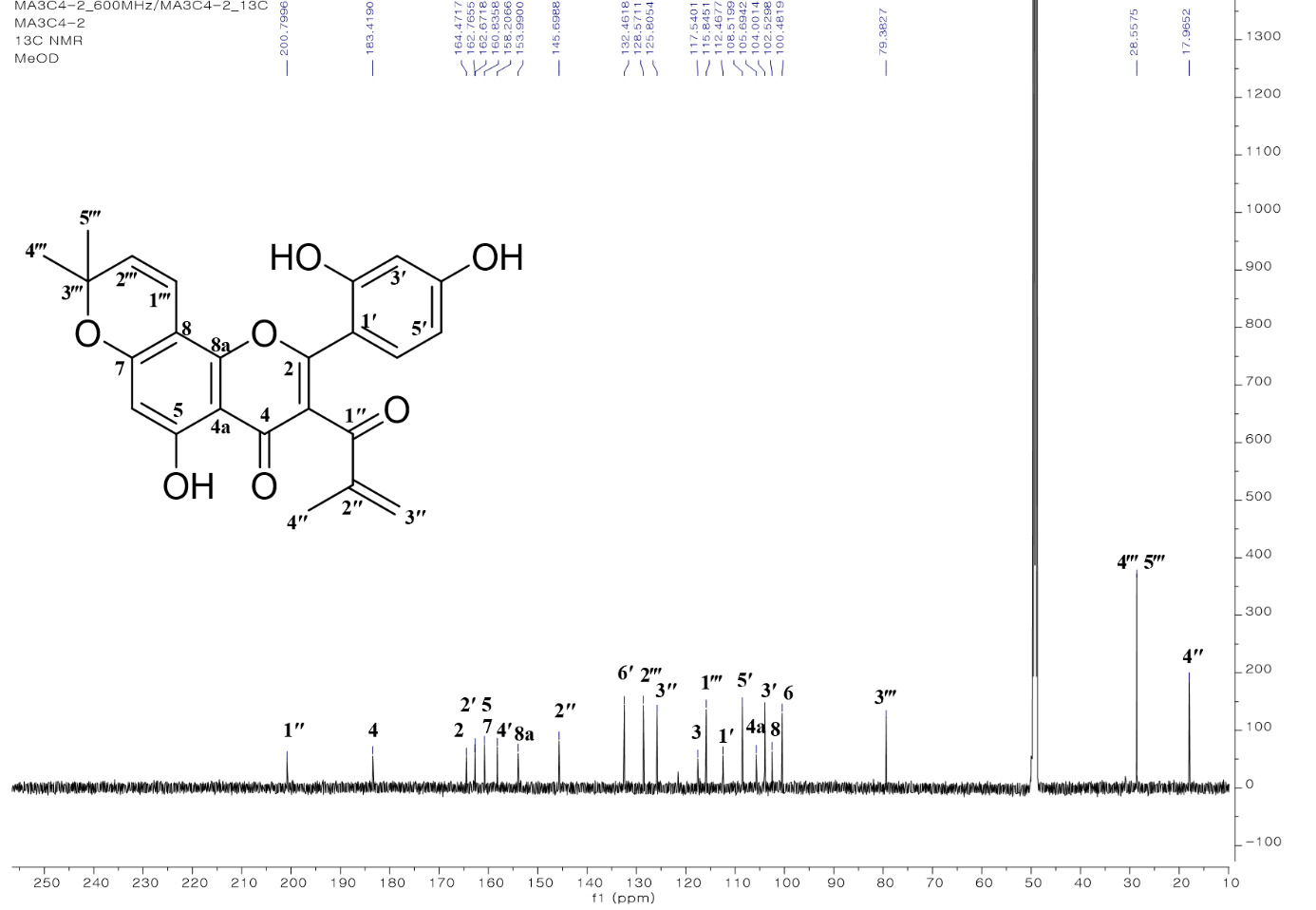
**

**Figure S2. ^1^H and ^13^C NMR spectrums of compound 1.**

**
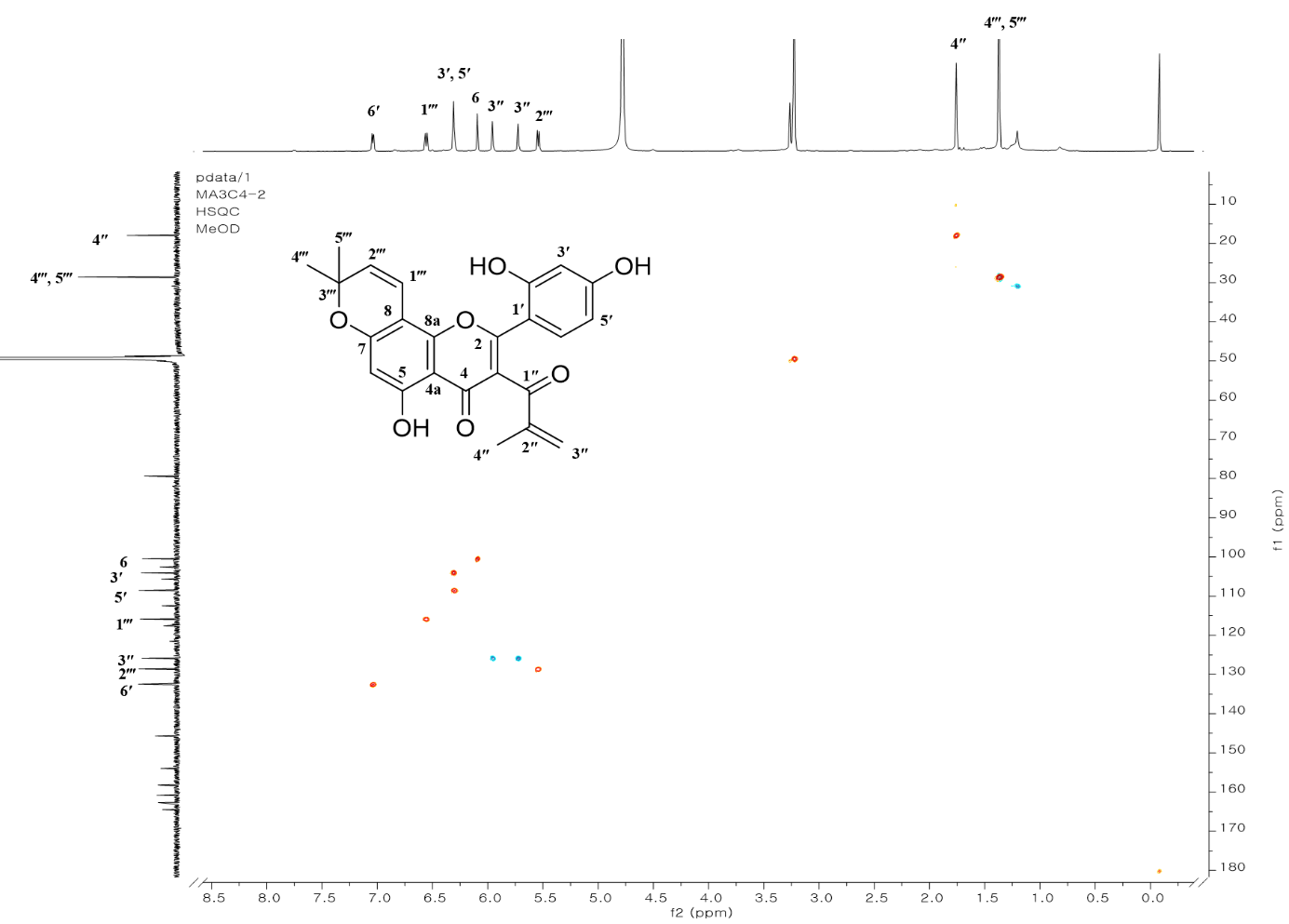
**

**
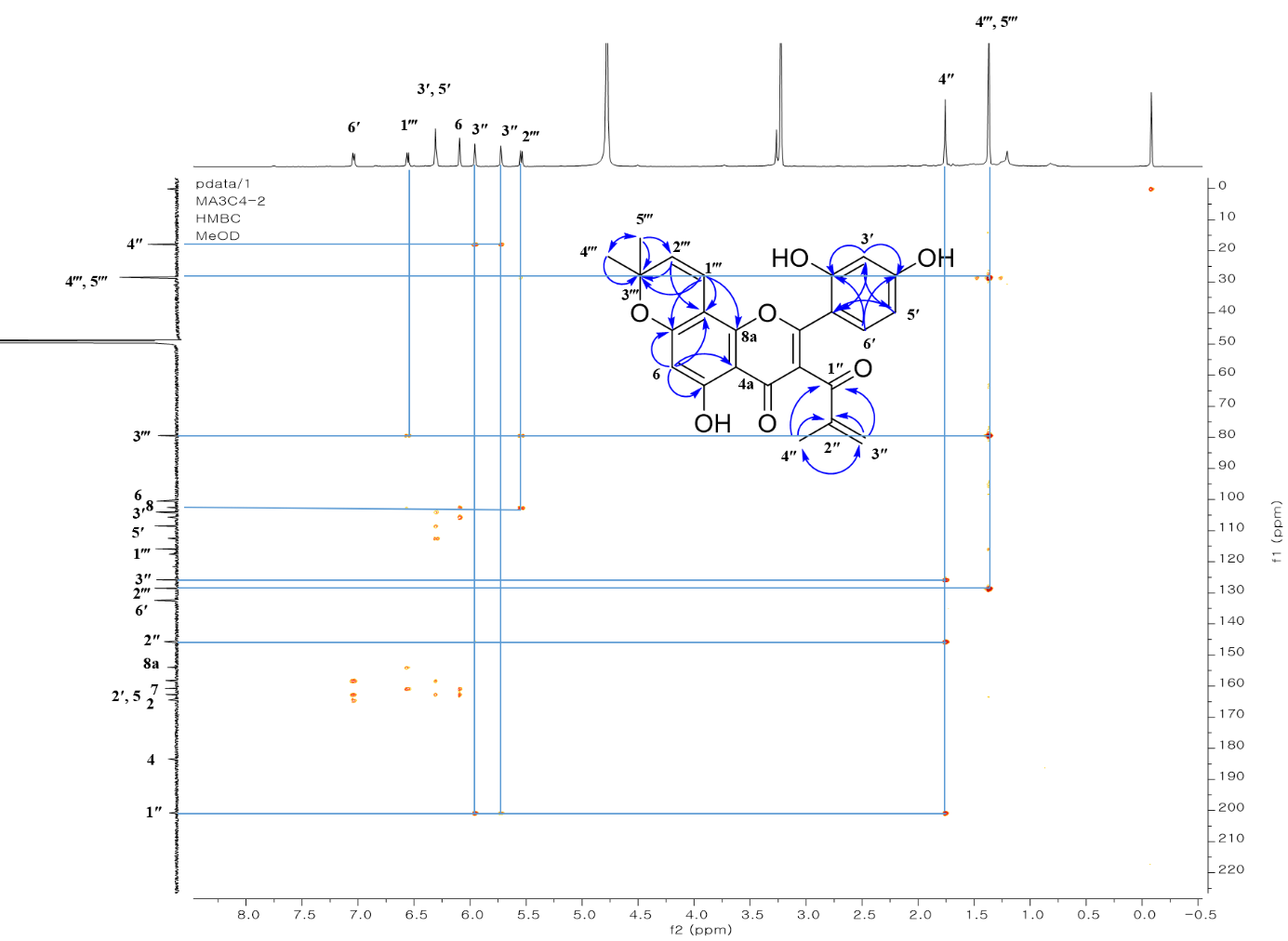
**

**Figure S3. HSQC and HMBC spectrums of compound 1.**


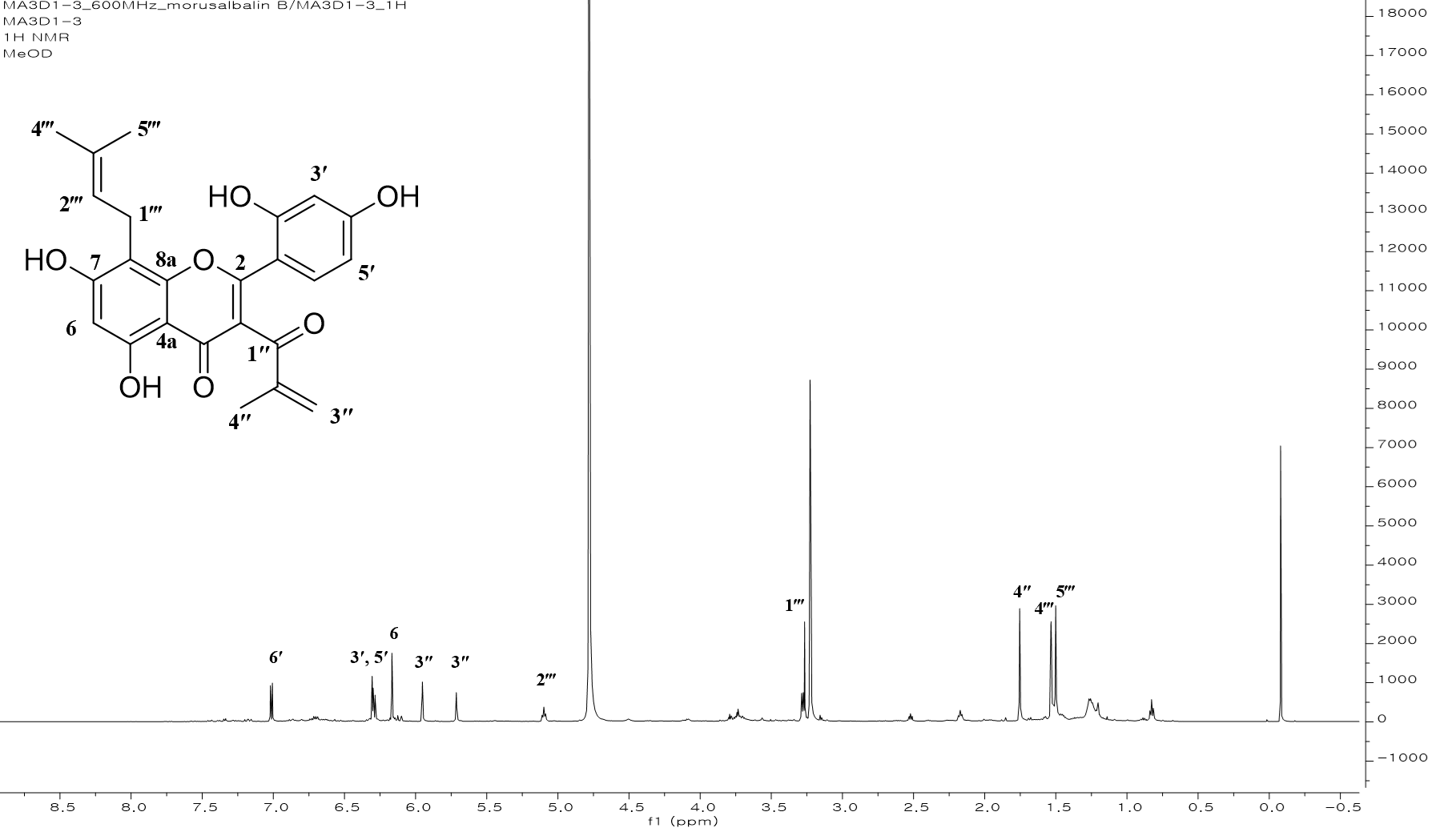


\
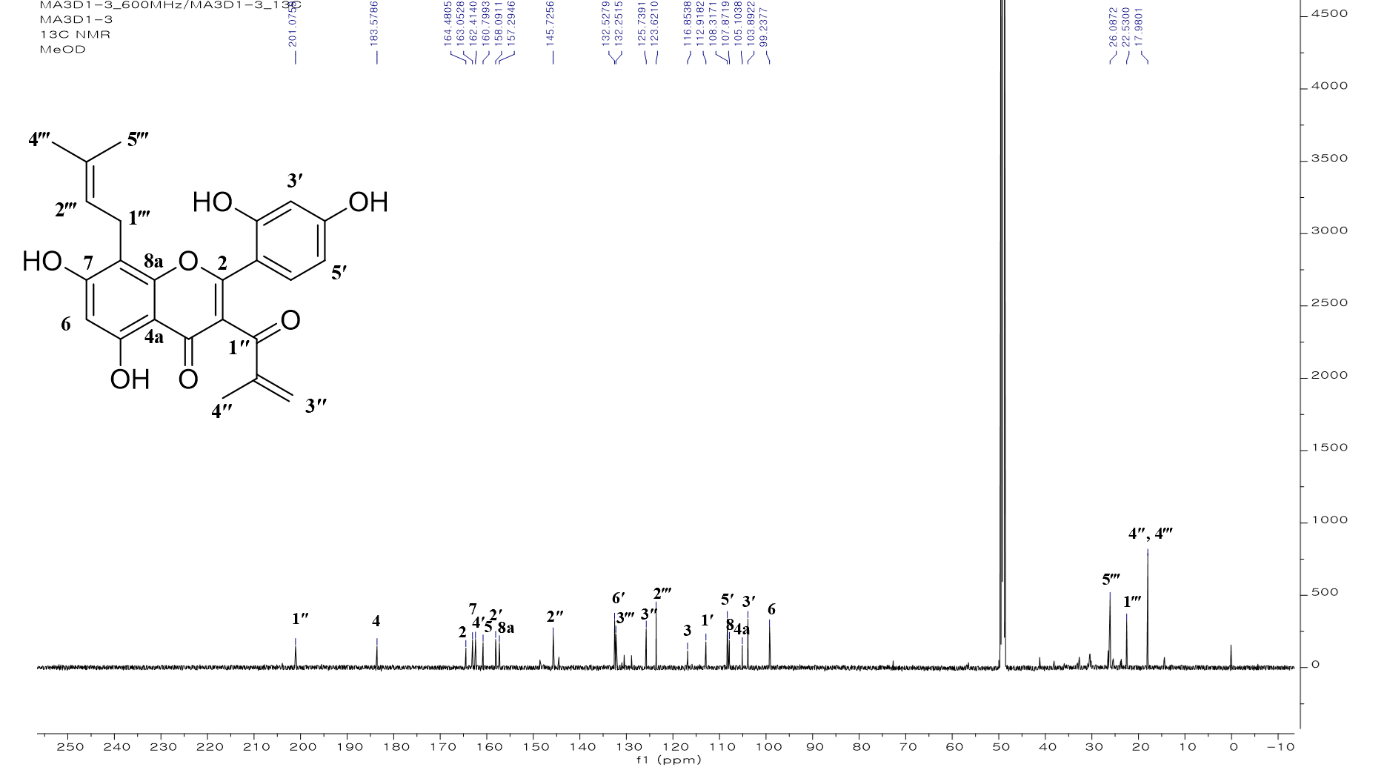


**Figure S4. ^1^H and ^13^C NMR spectrums of compound 5.**


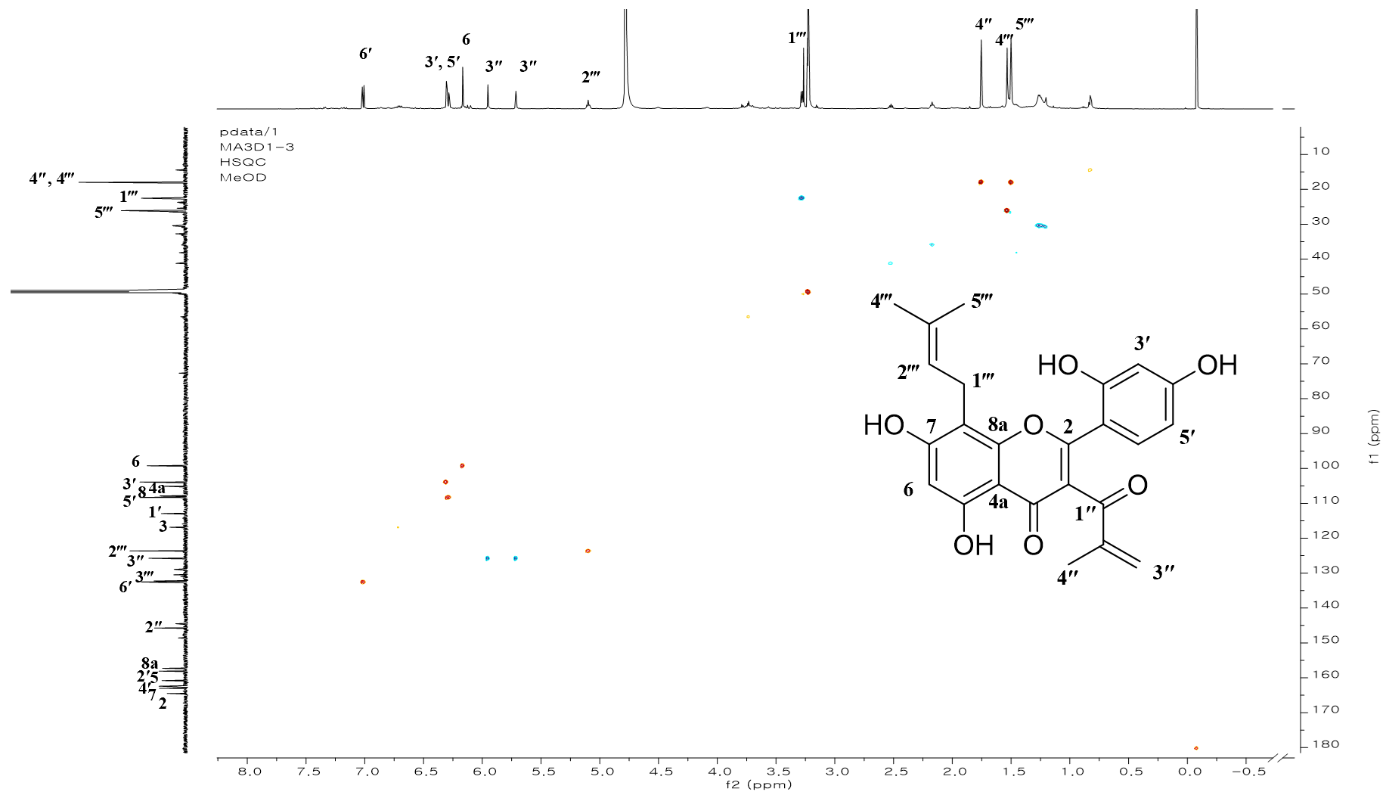


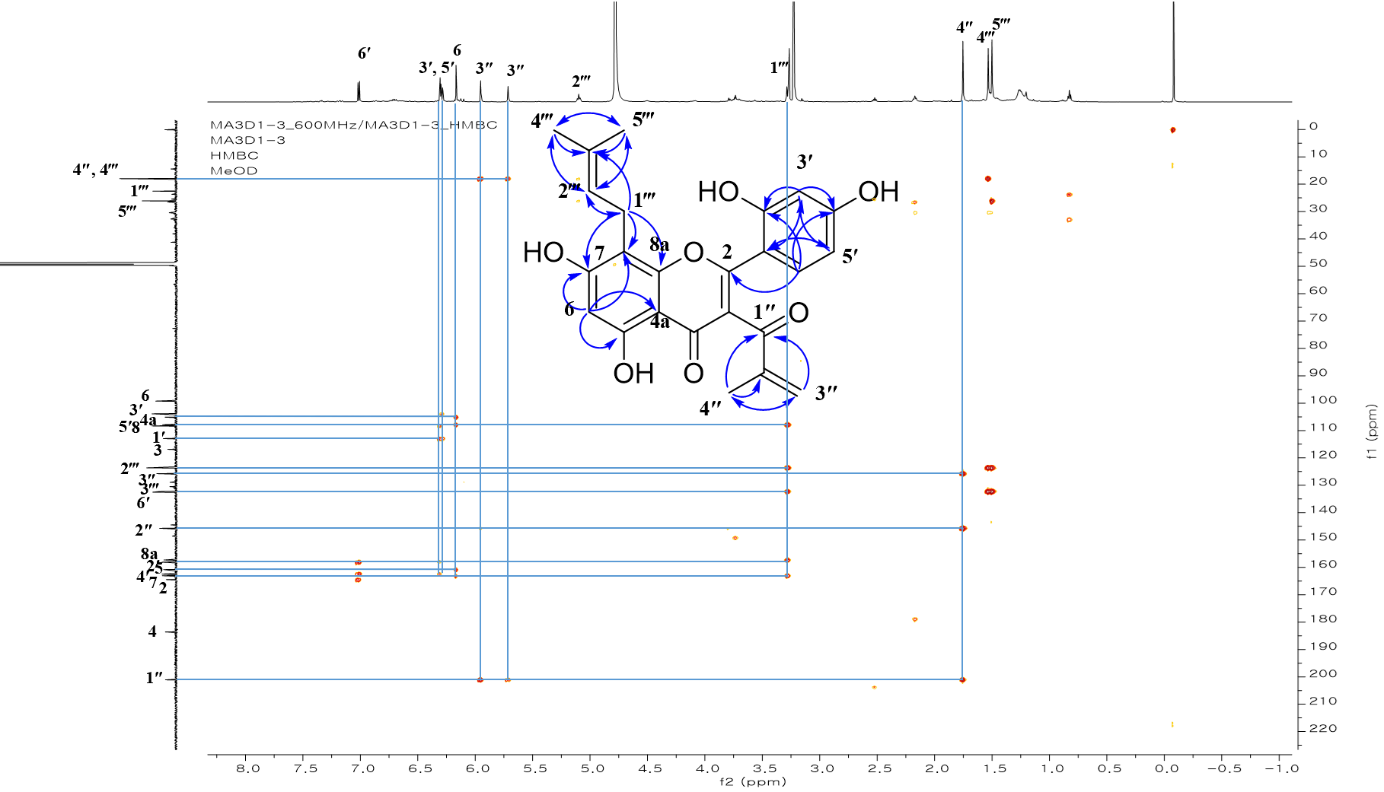


**Figure S5. HSQC and HMBC spectrums of compound 5.**


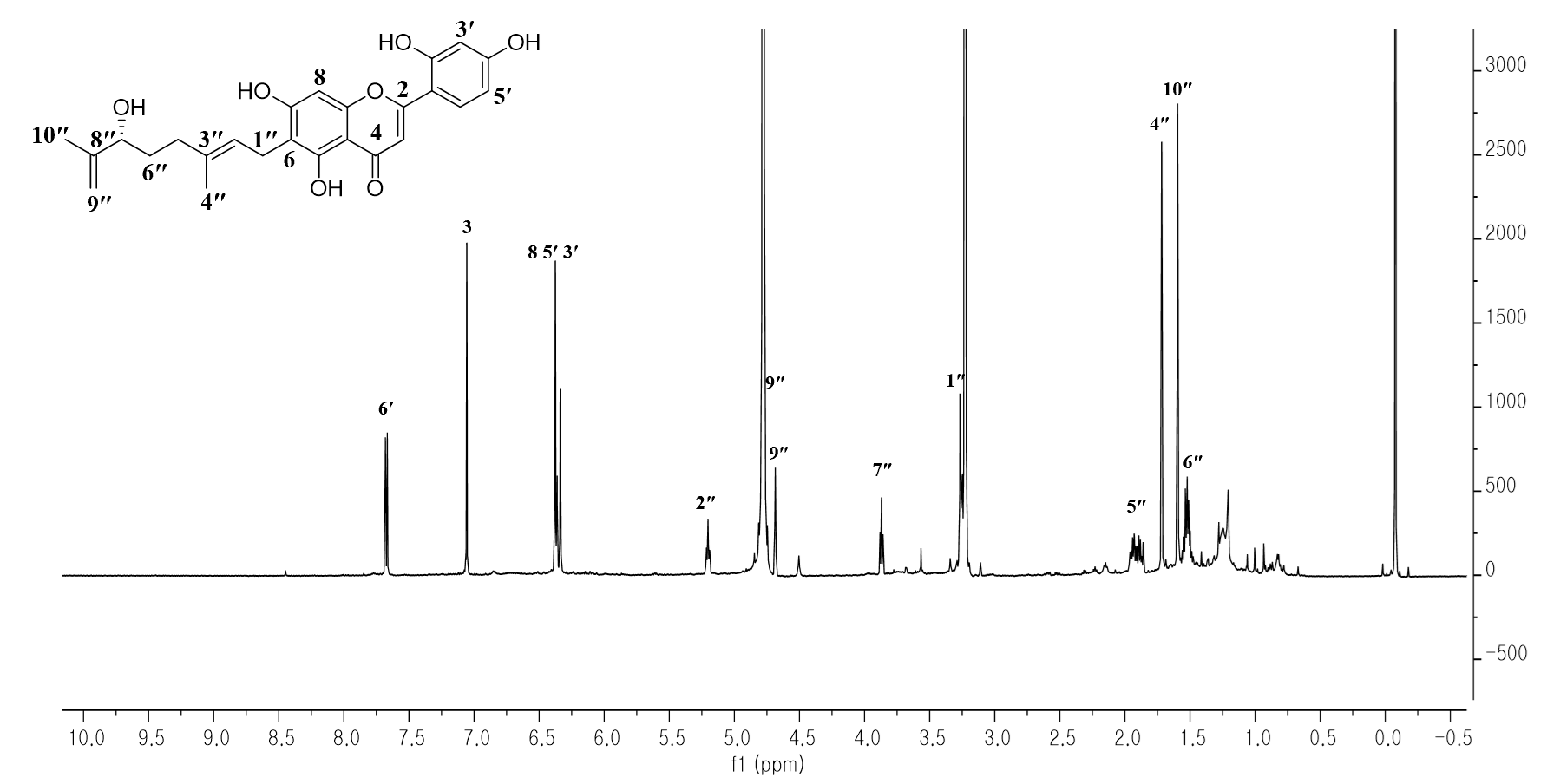


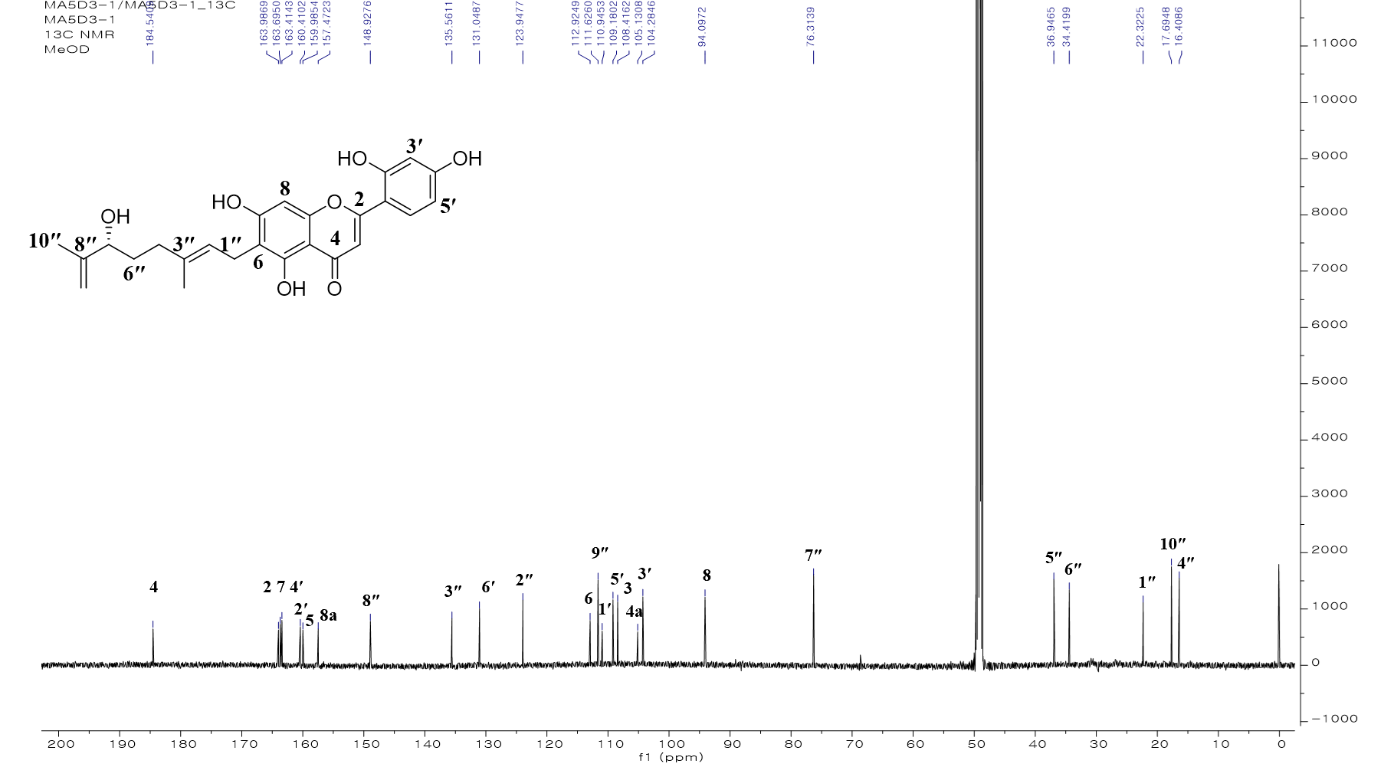


**Figure S6. ^1^H and ^13^C NMR spectrums of compound 10.**

**
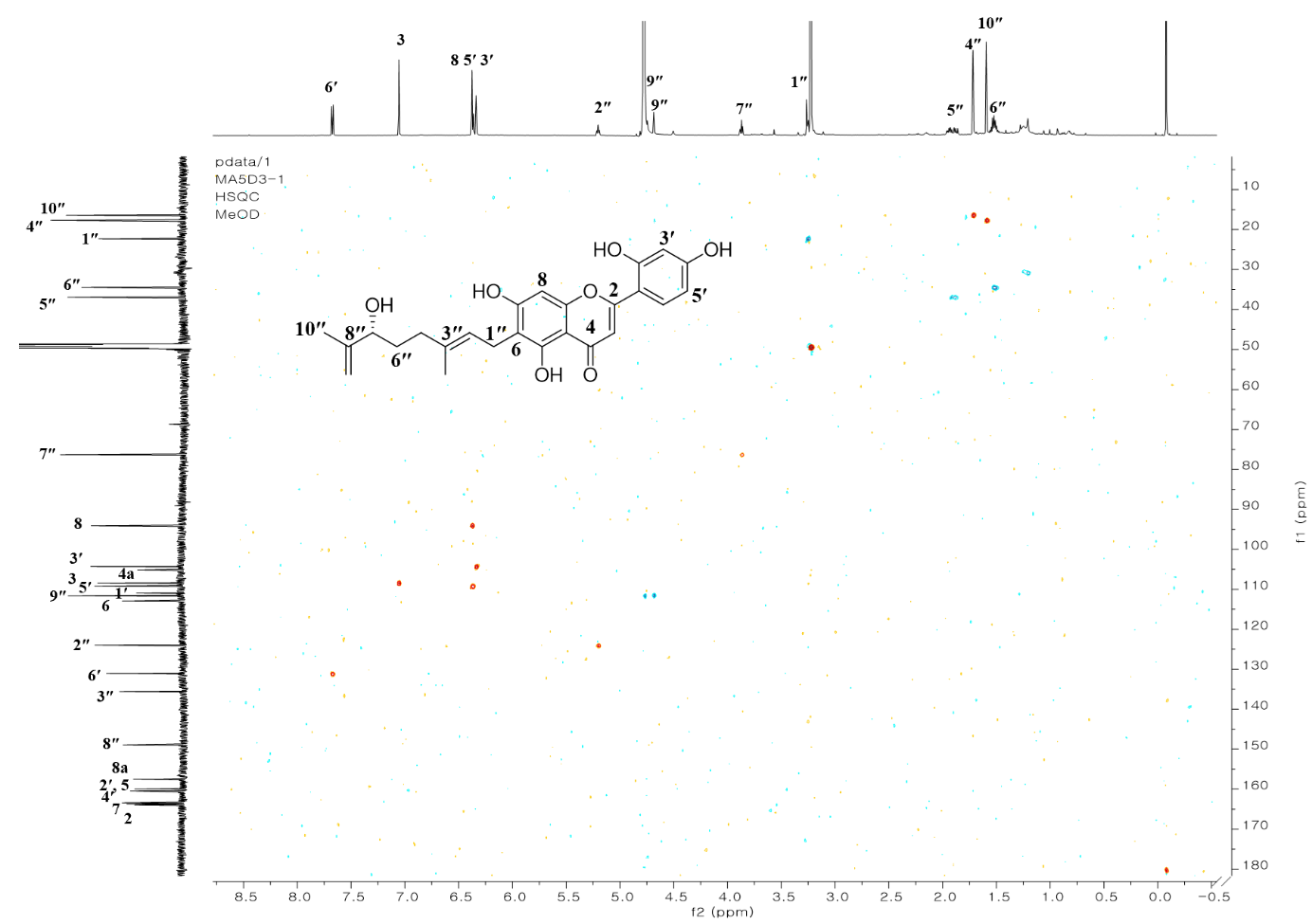
**

**
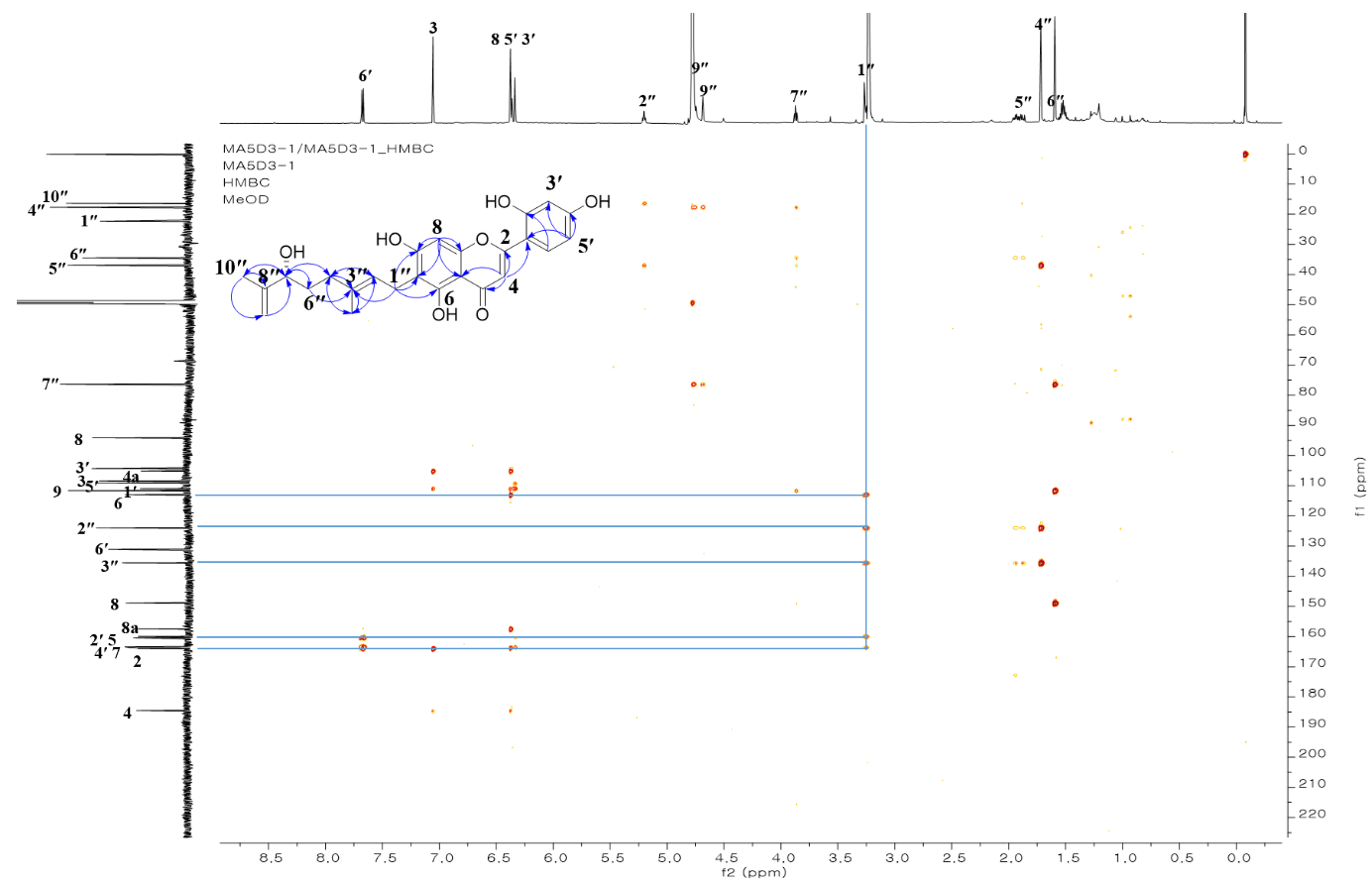
**

**Figure S7. HSQC and HBMC spectrums of compound 10.**


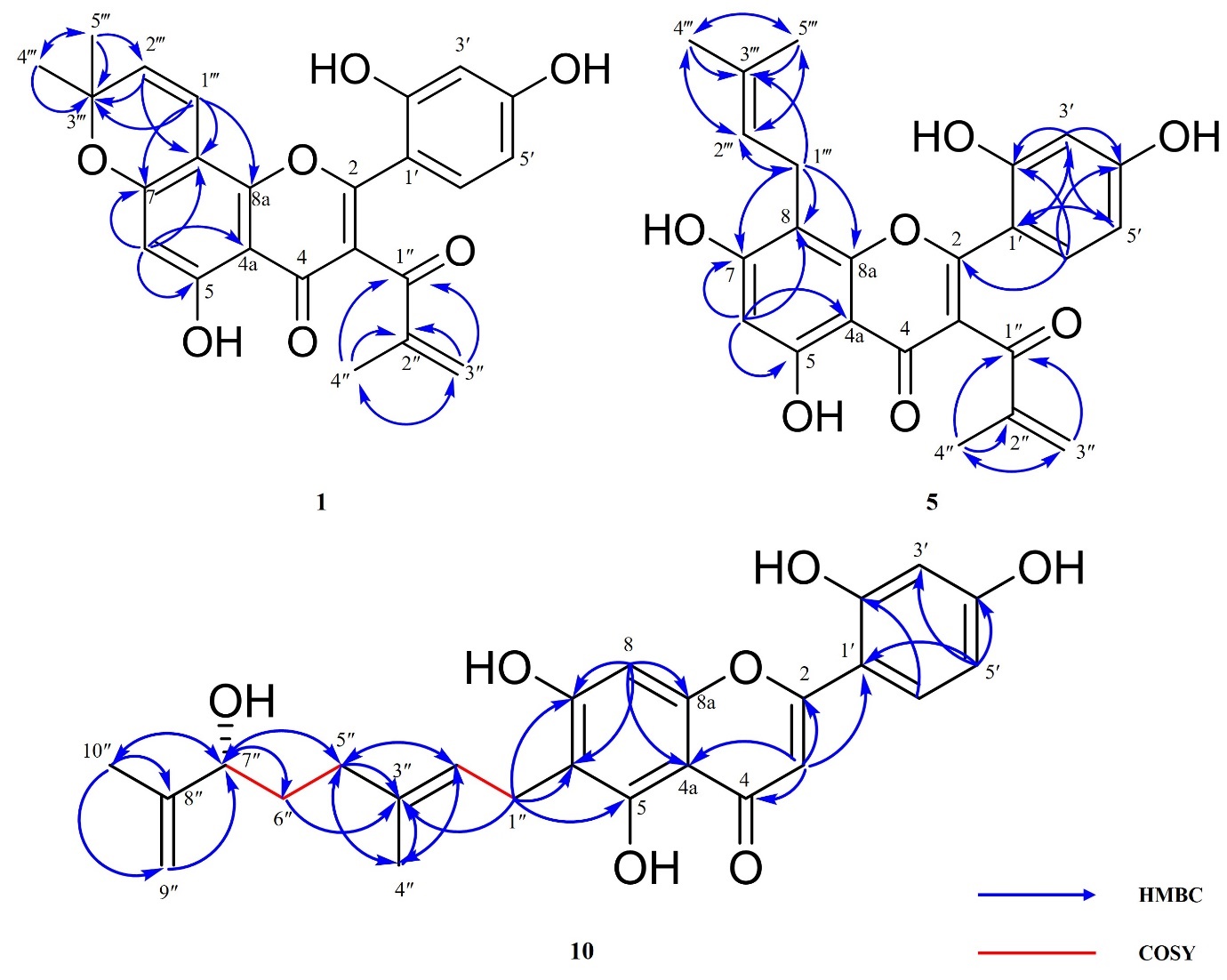


**Figure S8. Key HMBC and COSY correlation of compounds 1, 5 and 10.**

**Figure S9. HR-ESI-MS spectrum of morusalbalin A (1).**

**Figure S10. HR-ESI-MS spectrum of morusalbalin B (5).**

**Figure S11. HR-ESI-MS spectrum of morusalbalin C (10).**

**
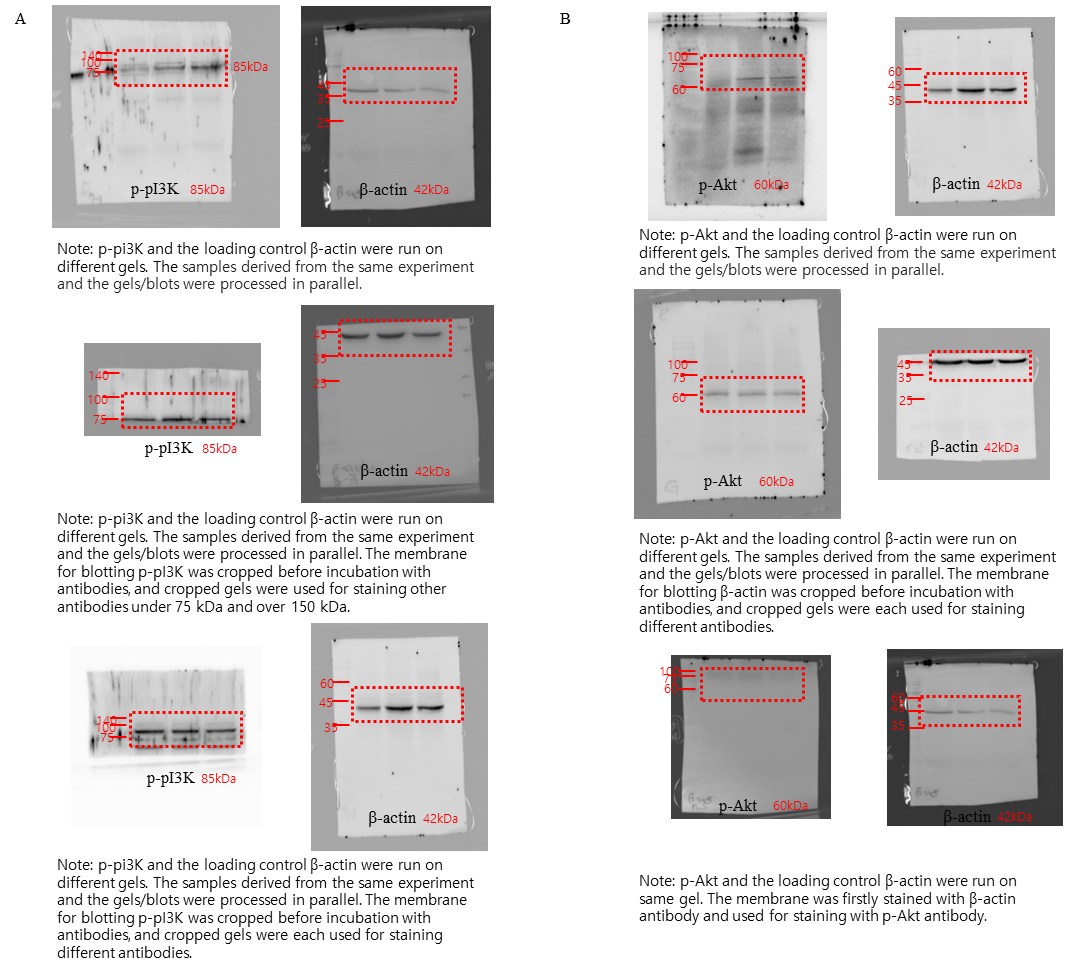

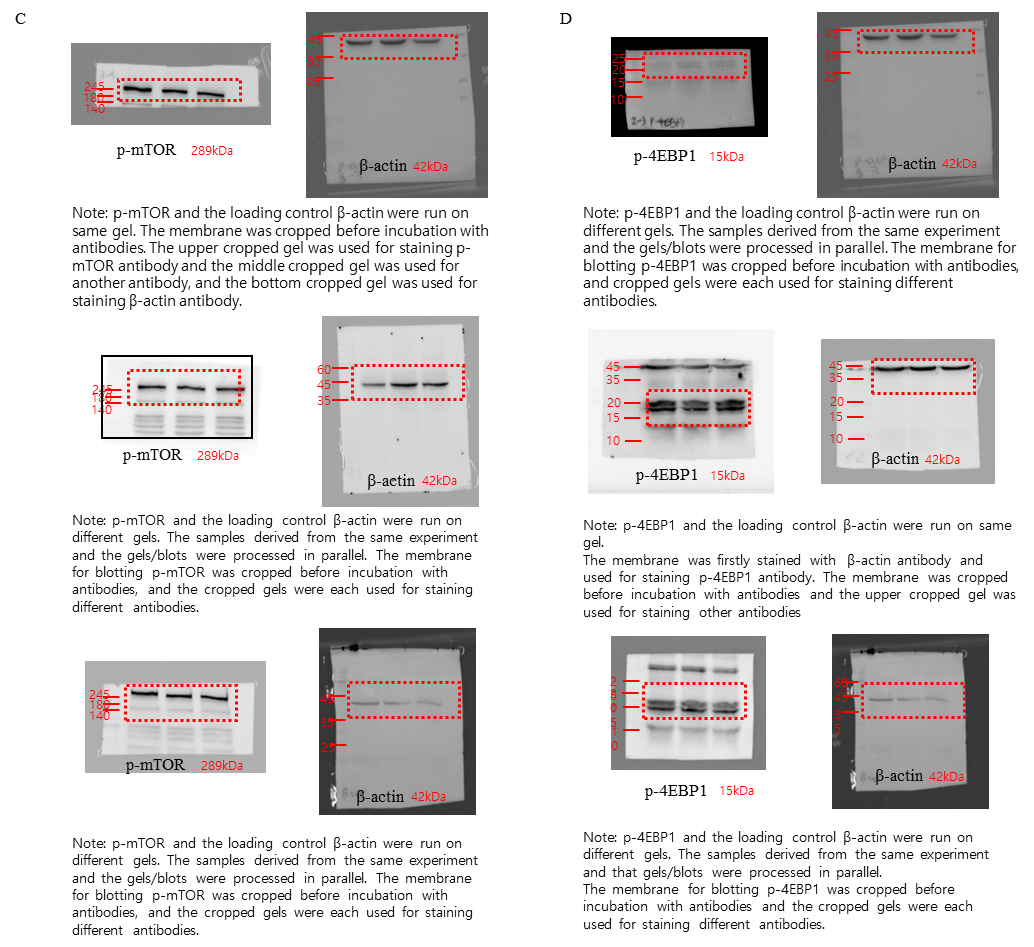
**

**
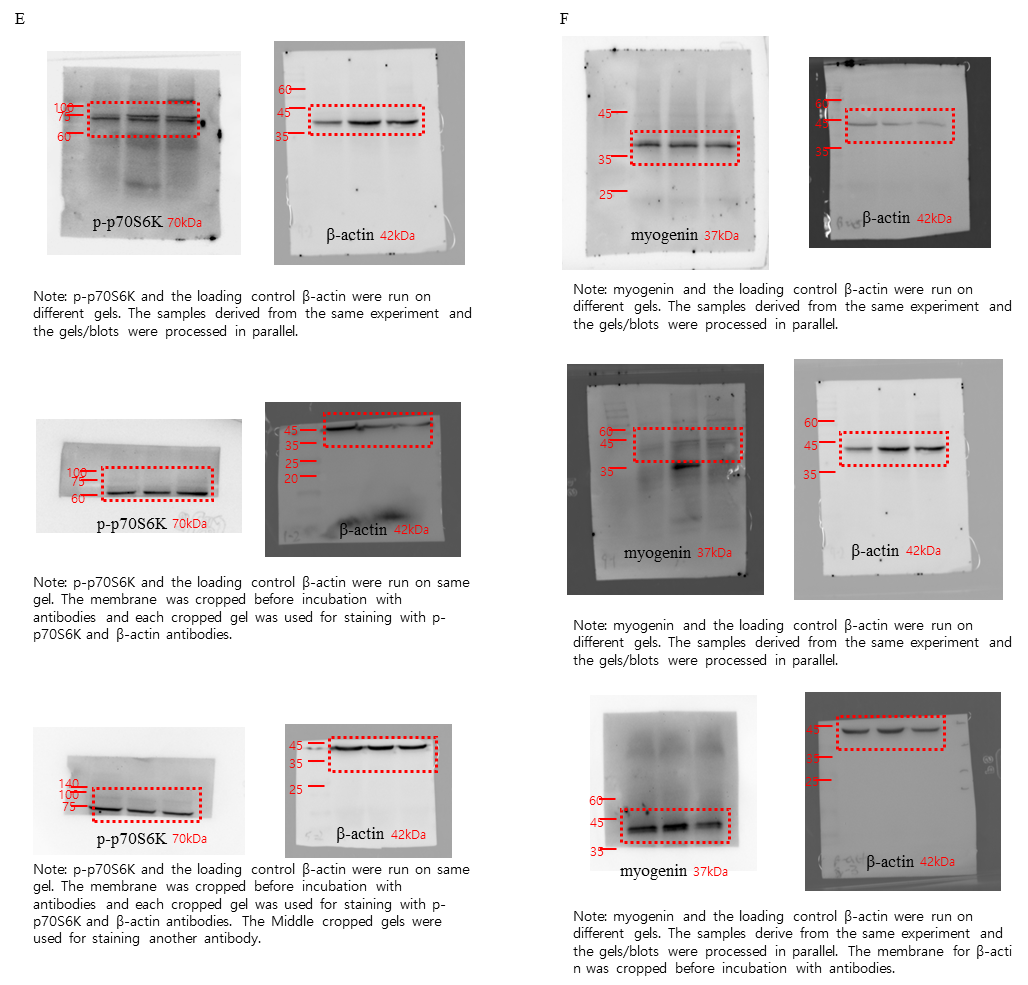
**

**
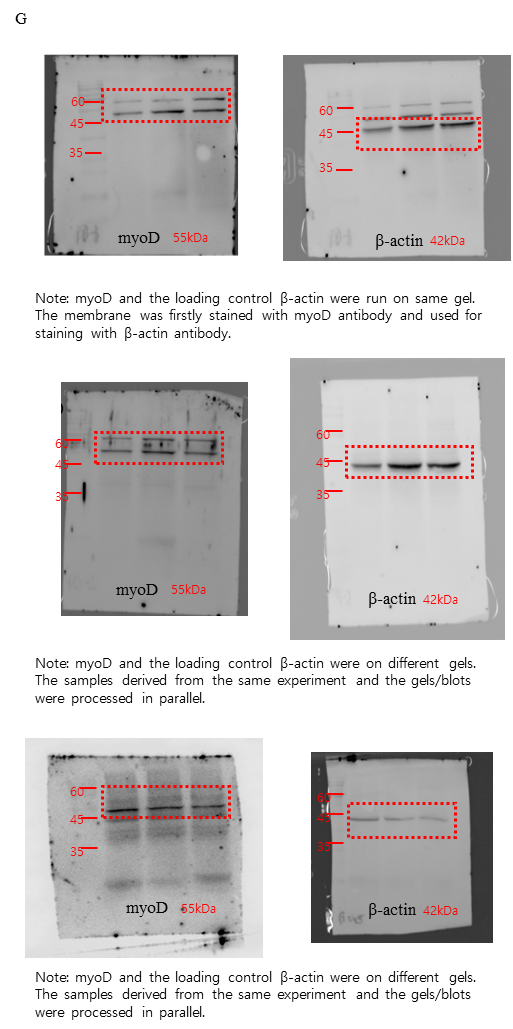
**

**Figure S12. Original blot of moracin E (21) effect on C2C12 cells.**

This includes the original blots of Figure 6A and triplicates of western blotting. Some of the membranes were cropped horizontally before incubating them with antibodies for the detection of multiple proteins.**
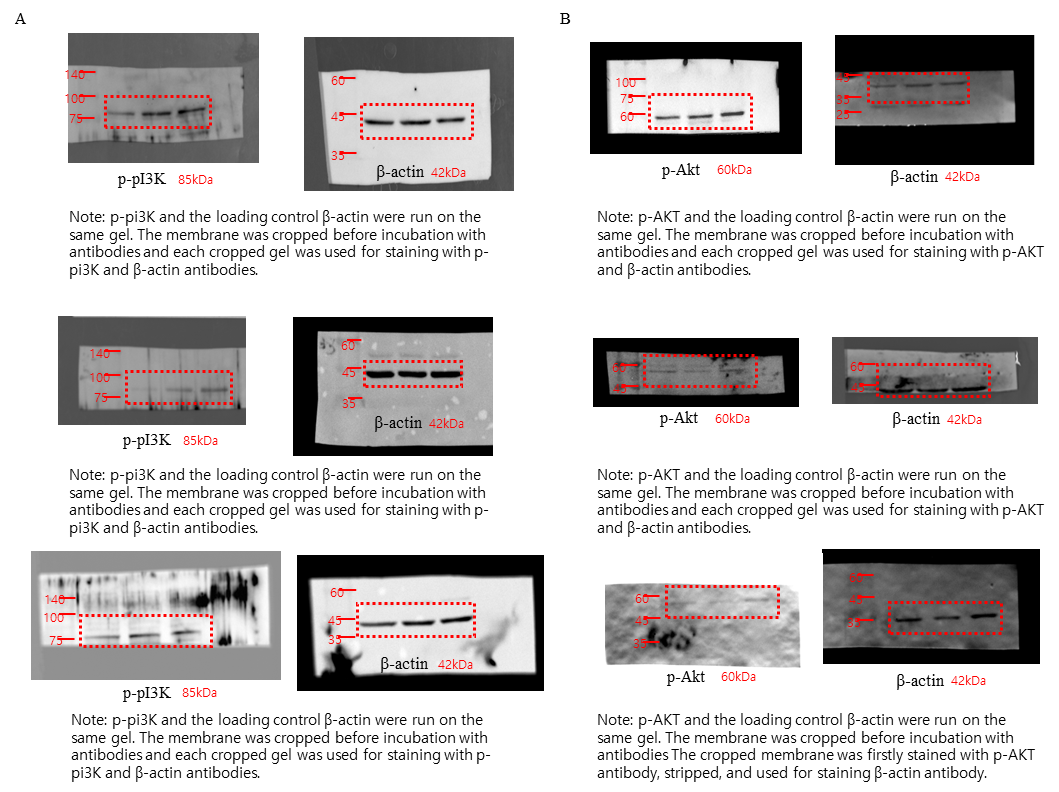
**

**
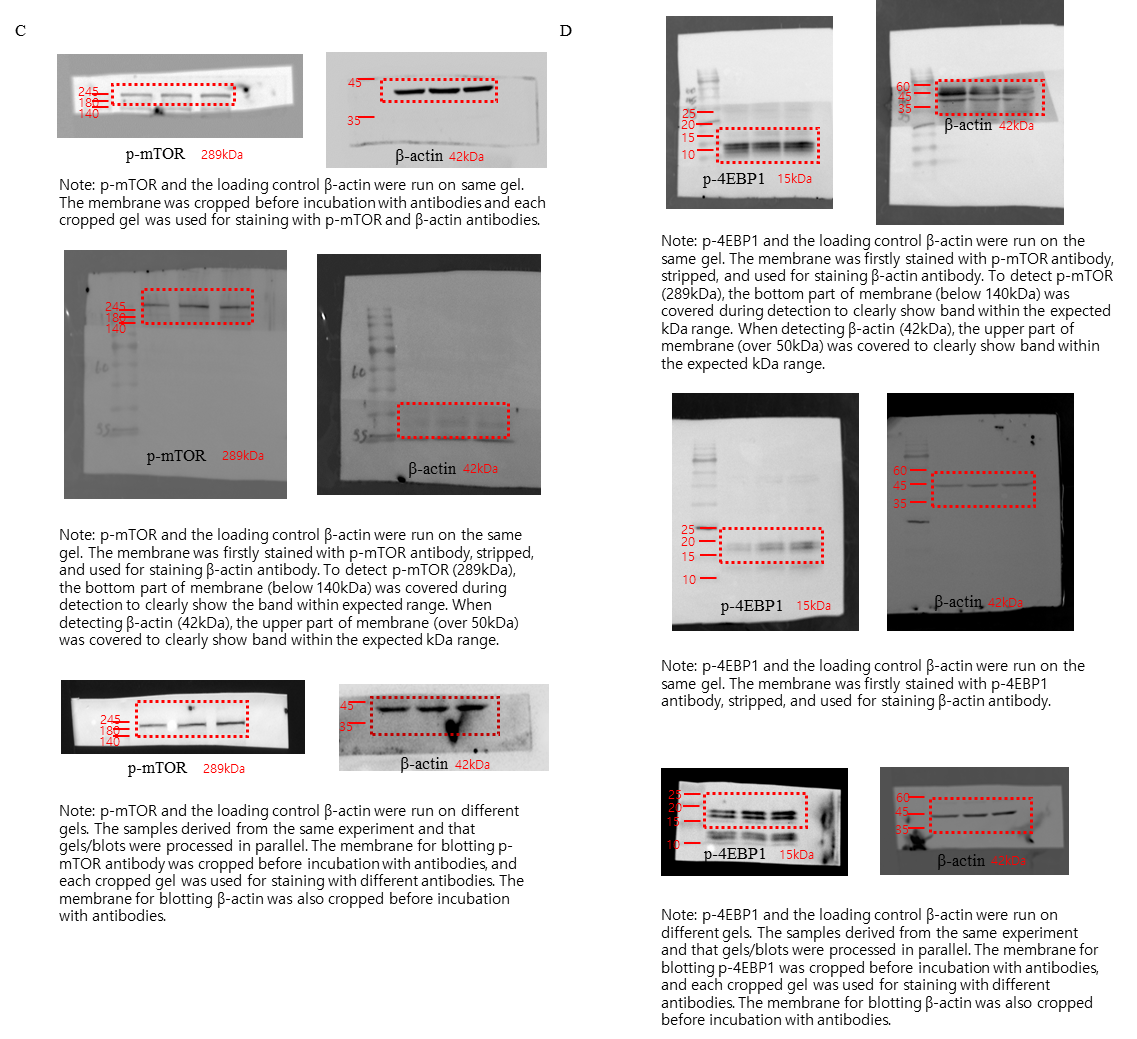
**

**
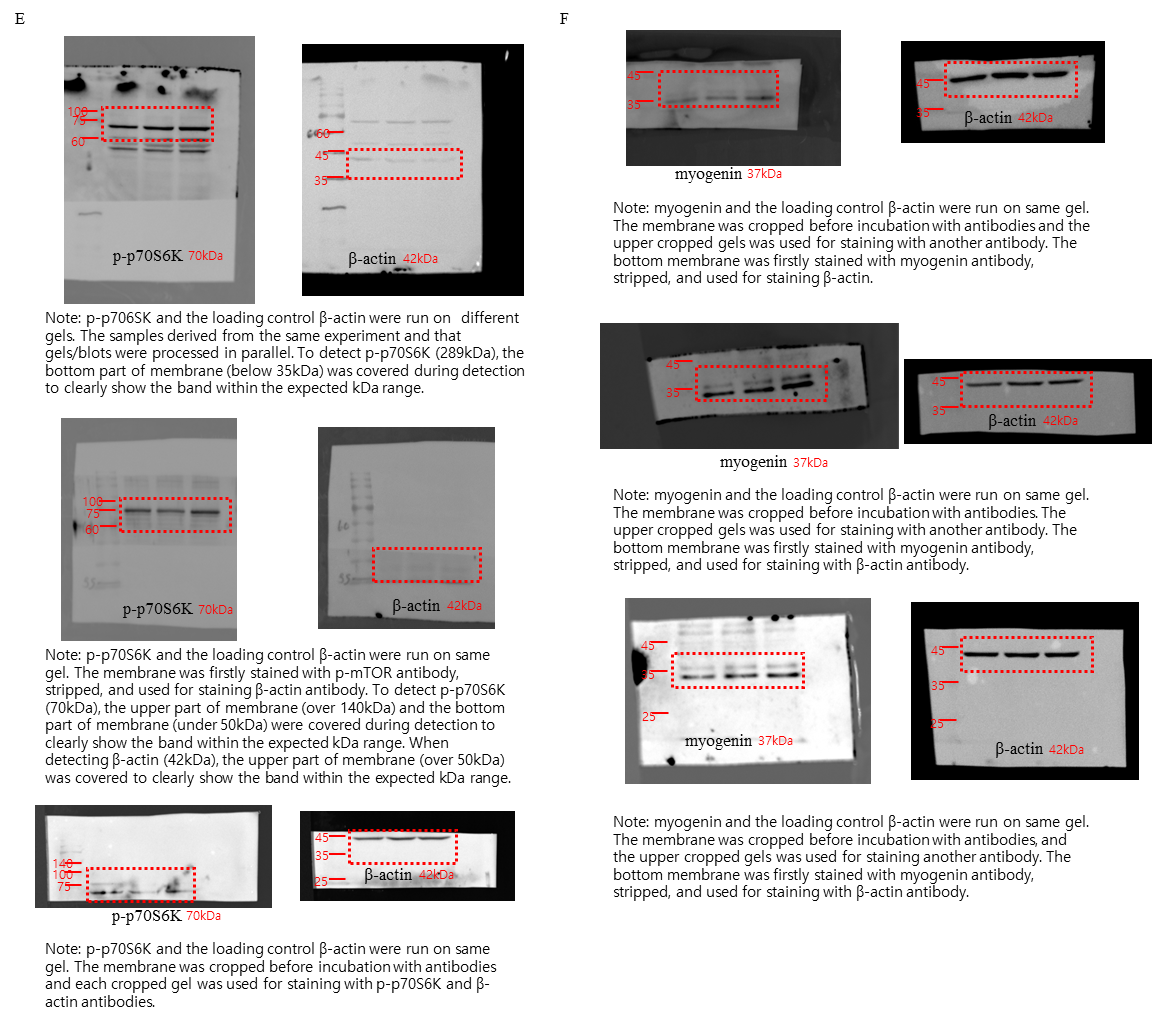
**

**
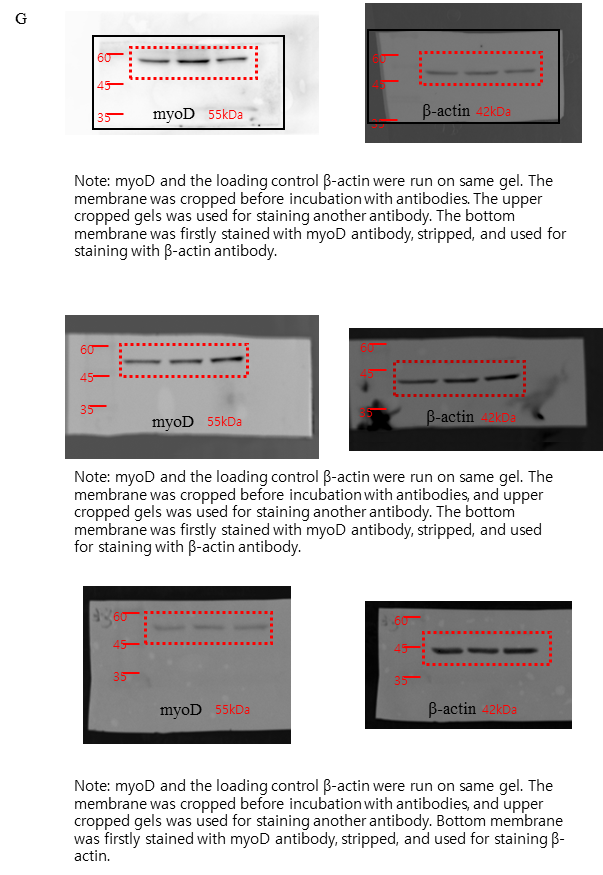
**

**Figure S13. Original blot of moracin M (23) effect on C2C12 cells.**

This includes the original blots of Figure 6I and triplicates of western blotting. Some of the membranes were cropped horizontally before incubating them with antibodies for the detection of multiple proteins.

**
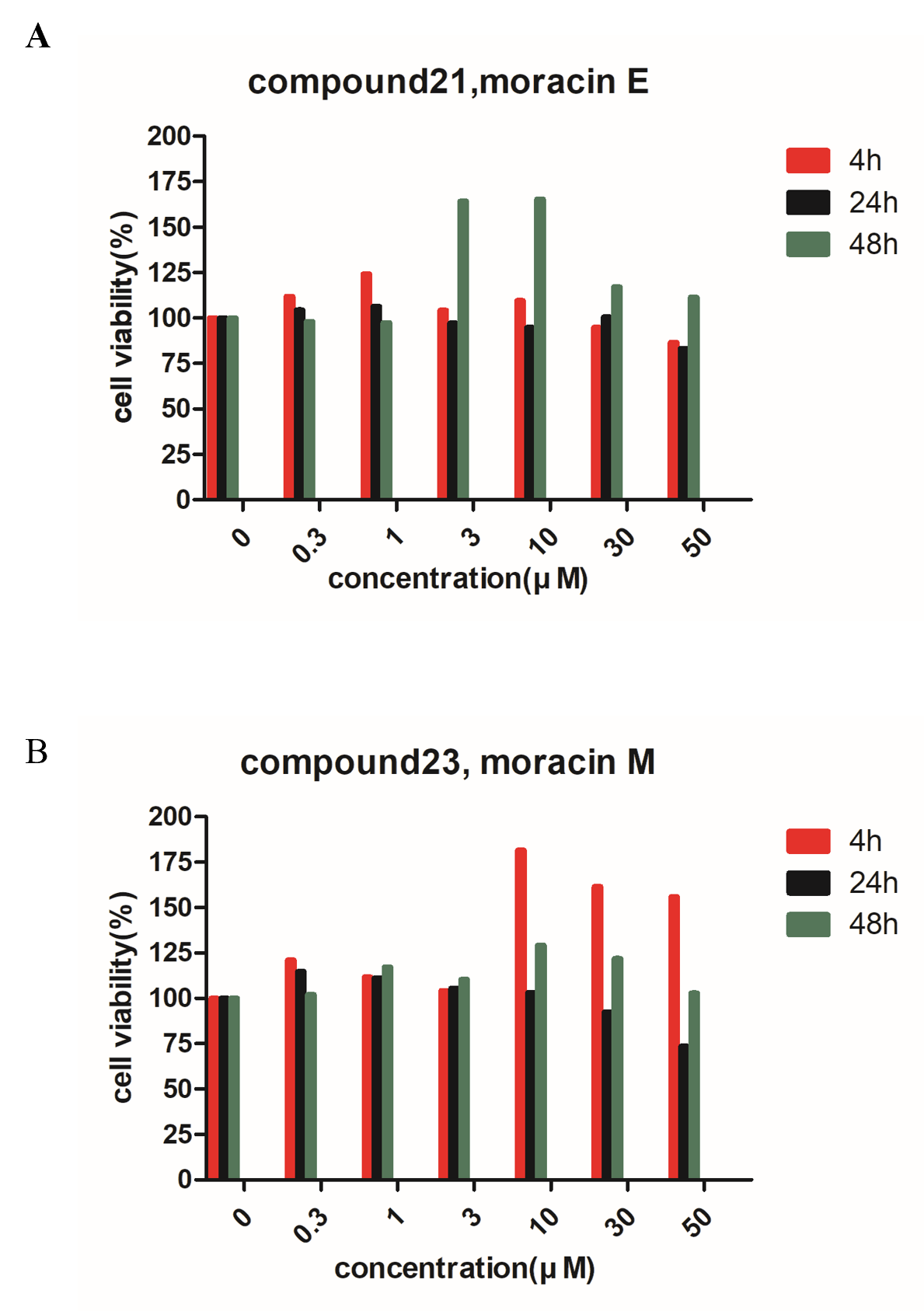
**

**Figure S14. Cell toxicity of moracin E (21) and moracin M (23)**

C2C12 cells were treated with moracin E and M at concentrations of 0, 0.3, 1, 3, 10, 30, and 50 µM for 4, 24, and 48 hours. Cell viability was determined using a CCK assay.

**Table S1. ^1^H and ^13^C NMR data of compounds 1, 5 and 10.**

| **Pos** | ***δ*_H_ ^a,c^** | ***δ*_C_ ^a,b^** | ***δ*_H_ ^a,c^** | ***δ*_C_ ^a,b^** | ***δ*_H_ ^a,c^** | ***δ*_C_ ^a,b^** |
| --- | --- | --- | --- | --- | --- | --- |
|  | **1** | | **5** | | **10** | |
| 2 | ‐ | 164.4 | ‐ | 164.5 | ‐ | 163.9 |
| 3 | ‐ | 117.5 | ‐ | 116.8 | 7.06 (s) | 108.4 |
| 4 | ‐ | 183.4 | ‐ | 183.5 | ‐ | 184.5 |
| 4a | ‐ | 105.6 | ‐ | 105.1 | ‐ | 105.1 |
| 5 | ‐ | 162.7 | ‐ | 160.7 | ‐ | 159.9 |
| 6 | 6.10 (s) | 100.4 | 6.17 (s) | 99.1 | ‐ | 112.9 |
| 7 | ‐ | 160.8 | ‐ | 163.0 | ‐ | 163.6 |
| 8 | ‐ | 102.5 | ‐ | 107.9 | ‐ | 94.0 |
| 8a | ‐ | 153.9 | ‐ | 157.3 | 6.37 (s) | 157.4 |
| 1′ | ‐ | 112.4 | ‐ | 112.9 | ‐ | 110.9 |
| 2′ | ‐ | 162.6 | ‐ | 158.0 | ‐ | 160.4 |
| 3′ | 6.31 (d, 2.8) | 104.0 | 6.31 (d, 2.2) | 103.8 | 6.34 (d, 2.3) | 104.2 |
| 4′ | ‐ | 158.2 | ‐ | 162.3 | ‐ | 163.4 |
| 5′ | 6.30 (dd, 2.8, 8.2) | 108.5 | 6.29 (dd, 2.3, 8.3) | 108.3 | 6.36 (dd, 2.3,8.8) | 109.1 |
| 6′ | 7.04 (d, 8.2) | 132.4 | 7.01 (d, 8.3) | 132.5 | 7.67 (d, 8.8) | 131.0 |
| 1″ | ‐ | 200.7 | ‐ | 201.0 | 3.26 (m) | 22.3 |
| 2″ | ‐ | 145.6 | ‐ | 145.7 | 5.20 (t, 7.2) | 123.9 |
| 3″ | 5.73 (s)  5.96 (s) | 125.8 | 5.71 (s)  5.95 (s) | 125.7 | ‐ | 135.5 |
| 4″ | 1.76 (s) | 17.9 | 1.75 (s) | 17.8 | 1.72 (s) | 16.4 |
| 5″ |  |  |  |  | 1.90 (m) | 36.9 |
| 6″ |  |  |  |  | 1.52 (m) | 34.4 |
| 7″ |  |  |  |  | 3.87 (t, 6.6) | 76.3 |
| 8″ |  |  |  |  | ‐ | 148.9 |
| 9″ |  |  |  |  | 4.69 (t, 1.8)  4.76 (overlap) | 111.6 |
| 10″ |  |  |  |  | 1.60 (s) | 17.6 |
| 1‴ | 6.56 (d, 10.1) | 115.8 | 3.28 (d, 7.3) | 22.5 |  |  |
| 2‴ | 5.54 (d, 10.2) | 128.5 | 5.10 (t, 7.3) | 123.6 |  |  |
| 3‴ | ‐ | 79.3 | ‐ | 132.2 |  |  |
| 4‴ | 1.37 (s) | 28.5 | 1.50 (s) | 17.9 |  |  |
| 5‴ | 1.37 (s) | 28.5 | 1.53 (s) | 25.0 |  |  |

^a^ Measured in CD_3_OD, ^b^150 MHz, ^c^600 MHz, Assignments were done by HSQC, HMBC and COSY experiment

**Table S2. QED and OB values of compounds 1‒43.**

| **No.** | **Compound** | **MW** | **ALOGP** | **HBA** | **HBD** | **PSA** | **ROTB** | **AROM** | **ALERTS** | **QED** | **OB** |
| --- | --- | --- | --- | --- | --- | --- | --- | --- | --- | --- | --- |
| 1 | Morusabalin A | 420.41 | 4.50 | 7 | 3 | 117.20 | 3 | 3 | 1 | 0.534 | TRUE |
| 2 | Morusin | 420.45 | 5.18 | 6 | 3 | 100.13 | 3 | 3 | 1 | 0.505 | TRUE |
| 3 | Albanin B | 436.45 | 4.58 | 7 | 4 | 120.36 | 4 | 3 | 1 | 0.442 | TRUE |
| 4 | 2-(2,4-Dihydroxyphenyl)-5-hydroxy-3-(2-hydroxy-3-methylbut-3-enyl)-8,8-dimethylpyrano[2,3-h]chromen-4-one | 436.45 | 4.58 | 7 | 4 | 120.36 | 4 | 3 | 1 | 0.442 | TRUE |
| 5 | Morusalbalin B | 422.43 | 4.86 | 7 | 4 | 128.20 | 5 | 3 | 2 | 0.341 | TRUE |
| 6 | Mornigrol E | 438.47 | 4.94 | 7 | 5 | 131.36 | 6 | 3 | 1 | 0.344 | TRUE |
| 7 | Mornigrol F | 438.47 | 4.94 | 7 | 5 | 131.36 | 6 | 3 | 1 | 0.344 | TRUE |
| 8 | Kuwanon C | 422.47 | 5.54 | 6 | 4 | 111.13 | 5 | 3 | 1 | 0.402 | TRUE |
| 9 | Mortatarin A | 438.47 | 5.36 | 7 | 4 | 123.66 | 5 | 3 | 2 | 0.312 | TRUE |
| 10 | Morusalbalin C | 438.47 | 4.81 | 7 | 5 | 131.36 | 7 | 3 | 1 | 0.335 | TRUE |
| 11 | (7″R)‐(-)‐6‐(7″‐hydroxy‐3″,8″‐dimethyl‐2″,8″‐octadien‐1″‐yl)apigenin | 422.47 | 5.28 | 6 | 4 | 111.13 | 7 | 3 | 1 | 0.386 | TRUE |
| 12 | Norartocarpetin | 286.24 | 2.03 | 6 | 4 | 111.13 | 1 | 3 | 0 | 0.544 | TRUE |
| 13 | Albanin A | 354.35 | 3.73 | 6 | 4 | 99.73 | 3 | 3 | 1 | 0.535 | TRUE |
| 14 | Cyclocommunol | 352.34 | 3.43 | 6 | 3 | 100.13 | 1 | 3 | 1 | 0.580 | TRUE |
| 15 | 3-*O*-methylquercetin | 316.26 | 2.06 | 7 | 4 | 120.36 | 2 | 3 | 1 | 0.534 | TRUE |
| 16 | Kaempferol 7-*O*-*β*-D-glucoside | 448.38 | -0.12 | 11 | 7 | 190.28 | 4 | 3 | 0 | 0.282 | FALSE |
| 17 | Quercetin 3-*O*-*β*-D-glucoside | 464.38 | -0.59 | 12 | 8 | 210.51 | 4 | 3 | 1 | 0.228 | FALSE |
| 18 | Rutin | 610.52 | -2.11 | 16 | 10 | 269.43 | 6 | 3 | 1 | 0.134 | FALSE |
| 19 | Isobavachalcone | 324.37 | 4.22 | 4 | 3 | 77.76 | 5 | 2 | 2 | 0.568 | TRUE |
| 20 | Moracin D | 308.33 | 3.85 | 4 | 2 | 62.83 | 1 | 3 | 0 | 0.717 | TRUE |
| 21 | Moracin E | 308.33 | 3.85 | 4 | 2 | 62.83 | 1 | 3 | 0 | 0.717 | TRUE |
| 22 | Moracin B | 286.28 | 3.00 | 5 | 2 | 72.06 | 3 | 3 | 0 | 0.774 | TRUE |
| 23 | Moracin M | 242.23 | 2.47 | 4 | 3 | 73.83 | 1 | 3 | 0 | 0.612 | TRUE |
| 24 | Moracin M 6-*O*-*β*-D-glucoside | 404.37 | 0.28 | 9 | 6 | 152.98 | 4 | 3 | 0 | 0.360 | FALSE |
| 25 | Moracin M-3'-*O*-*β*-D-glucoside | 464.38 | -0.59 | 12 | 8 | 210.51 | 4 | 3 | 1 | 0.228 | FALSE |
| 26 | Scopoletin | 192.17 | 1.94 | 4 | 1 | 59.67 | 1 | 2 | 1 | 0.701 | TRUE |
| 27 | Scopolin | 354.31 | -0.20 | 9 | 4 | 138.82 | 4 | 2 | 1 | 0.550 | FALSE |
| 28 | Cichoriin | 340.28 | -0.72 | 9 | 5 | 149.82 | 3 | 2 | 1 | 0.456 | FALSE |
| 29 | Umbelliferone-7-*O*-*α*-rhamnopyranosyl-(1-6)-*β*-D-glucoside | 470.42 | -1.77 | 12 | 6 | 188.51 | 5 | 2 | 1 | 0.270 | FALSE |
| 30 | Oxyresveratrol | 244.24 | 2.08 | 4 | 4 | 80.92 | 2 | 2 | 1 | 0.610 | TRUE |
| 31 | Oxyresveratrol 4-*O*-*β*-D-glucoside | 406.38 | 0.00 | 9 | 7 | 160.07 | 5 | 2 | 1 | 0.339 | FALSE |
| 32 | Oxyresveratrol 3'-*O*-*β*-D-glucoside | 406.38 | 0.00 | 9 | 7 | 160.07 | 5 | 2 | 1 | 0.339 | FALSE |
| 33 | Dadahol A | 698.71 | 6.46 | 12 | 4 | 170.44 | 18 | 4 | 2 | 0.066 | FALSE |
| 34 | Dadahol B | 668.69 | 6.35 | 11 | 4 | 161.21 | 17 | 4 | 2 | 0.073 | FALSE |
| 35 | 4-hydroxybenzaldehyde | 122.12 | 1.52 | 2 | 1 | 37.30 | 1 | 1 | 1 | 0.573 | TRUE |
| 36 | 2,4-dihydroxybenzaldehyde | 138.12 | 1.02 | 3 | 2 | 57.53 | 1 | 1 | 1 | 0.569 | TRUE |
| 37 | C-veratroylglycol | 212.20 | 0.69 | 5 | 3 | 86.99 | 4 | 1 | 0 | 0.683 | TRUE |
| 38 | 3,4-dimethoxyphenyl-*β*-D-glycoside | 316.30 | -0.65 | 8 | 4 | 117.84 | 5 | 1 | 0 | 0.566 | TRUE |
| 39 | 3,4,5-trimethoxyphenyl-*β*-D-glycoside | 346.33 | -0.58 | 9 | 4 | 127.07 | 6 | 1 | 0 | 0.535 | FALSE |
| 40 | Kelampayoside A | 478.44 | -1.58 | 13 | 6 | 185.99 | 9 | 1 | 0 | 0.239 | FALSE |
| 41 | Icariside B_1_ | 386.44 | 0.31 | 8 | 5 | 136.68 | 4 | 0 | 1 | 0.436 | FALSE |
| 42 | Nicotinic acid | 123.11 | 0.75 | 3 | 1 | 50.19 | 1 | 1 | 0 | 0.599 | TRUE |
| 43 | Adenosine | 267.24 | -2.37 | 7 | 4 | 139.54 | 2 | 2 | 0 | 0.476 | TRUE |

**Table S3. List of expected active compounds.**

| **No.** | **Compound** | **QED** | **OB** |
| --- | --- | --- | --- |
| 1 | Morusalbain A (**1**) | 0.534 | TRUE |
| 2 | Morusin (**2**) | 0.505 | TRUE |
| 3 | Albanin B (**3**) | 0.442 | TRUE |
| 4 | 2-(2,4-Dihydroxyphenyl)-5-hydroxy-3-(2-hydroxy-3-methylbut-3-enyl)-8,8-dimethylpyrano[2,3-h]chromen-4-one (**4**) | 0.442 | TRUE |
| 5 | Morusalbalin B (**5**) | 0.341 | TRUE |
| 6 | Mornigrol E (**6**) | 0.344 | TRUE |
| 7 | Mornigrol F (**7**) | 0.344 | TRUE |
| 8 | Kuwanon C (**8**) | 0.402 | TRUE |
| 9 | Mortatarin A (**9**) | 0.312 | TRUE |
| 10 | Morusalbalin C (**10**) | 0.335 | TRUE |
| 11 | (7″R)-(−)-6-(7″-hydroxy-3″,8″-dimethyl-2″,8″-octadien-1″-yl)apigenin (**11**) | 0.386 | TRUE |
| 12 | Norartocarpetin (**12**) | 0.544 | TRUE |
| 13 | Albanin A (**13**) | 0.535 | TRUE |
| 14 | Cyclocommunol (**14**) | 0.580 | TRUE |
| 15 | 3-*O*-methylquercetin (**15**) | 0.534 | TRUE |
| 16 | Isobavachalcone (**19**) | 0.568 | TRUE |
| 17 | Moracin D (**20**) | 0.717 | TRUE |
| 18 | Moracin E (**21**) | 0.717 | TRUE |
| 19 | Moracin B (**22**) | 0.774 | TRUE |
| 20 | Moracin M (**23**) | 0.612 | TRUE |
| 21 | Scopoletin (**27**) | 0.701 | TRUE |
| 22 | Oxyresveratrol (**30**) | 0.610 | TRUE |
| 23 | 4-hydroxybenzaldehyde (**35**) | 0.573 | TRUE |
| 24 | 2,4-dihydroxybenzaldehyde (**36**) | 0.569 | TRUE |
| 25 | C-veratroylglycol (**37**) | 0.683 | TRUE |
| 26 | 3,4-dimethoxyphenyl-*β*-D-glycoside (**38**) | 0.566 | TRUE |
| 27 | Nicotinic acid (**42**) | 0.599 | TRUE |
| 28 | Adenosine (**43**) | 0.476 | TRUE |

**Table S4. List of potential targets.**

| **No.** | **Uniprot ID** | **Gene** | **Target** | **Protein class** |
| --- | --- | --- | --- | --- |
| 1 | P23443 | RPS6KB1 | ribosomal protein S6 kinase B1 | Kinase |
| 2 | P53779 | MAPK10 | mitogen-activated protein kinase 10 | Kinase |
| 3 | P28845 | HSD11B1 | hydroxysteroid 11-beta dehydrogenase 1 | - |
| 4 | P19838 | NFKB1 | nuclear factor kappa B subunit 1 | Transcription factor |
| 5 | P42345 | MTOR | mechanistic target of rapamycin kinase | Kinase |
| 6 | P12821 | ACE | angiotensin I converting enzyme | Enzyme |
| 7 | P45379 | TNNT2 | troponin T2, cardiac type | Cellular structure |
| 8 | P19429 | TNNI3 | troponin I3, cardiac type | Cellular structure |
| 9 | P31749 | AKT1 | AKT serine/threonine kinase 1 | Kinase |
| 10 | P01375 | TNF | tumor necrosis factor | Signaling |
| 11 | P51451 | BLK | BLK proto-oncogene, Src family tyrosine kinase | Kinase |
| 12 | P21554 | CNR1 | cannabinoid receptor 1 | G-protein coupled receptor |
| 13 | Q9Y618 | NCOR2 | nuclear receptor corepressor 2 | Transcription factor |
| 14 | Q92731 | ESR2 | estrogen receptor 2 | Nuclear receptor |
| 15 | P03372 | ESR1 | estrogen receptor 1 | Nuclear receptor |
| 16 | P02766 | TTR | transthyretin | Transporter |
| 17 | P08069 | IGF1R | insulin like growth factor 1 receptor | Kinase |
| 18 | P17936 | IGFBP3 | insulin like growth factor binding protein 3 | Enzyme modulator |
| 19 | P11474 | ESRRA | estrogen related receptor alpha | Nuclear receptor |
| 20 | P10275 | AR | androgen receptor | Nuclear receptor |
| 21 | P48736 | PIK3CG | phosphatidylinositol-4,5-bisphosphate 3-kinase catalytic subunit gamma | Kinase |
| 22 | P07451 | CA3 | carbonic anhydrase 3 | - |
| 23 | P08254 | MMP3 | matrix metallopeptidase 3 | Enzyme |
| 24 | P29275 | ADORA2B | adenosine A2b receptor | G-protein coupled receptor |

**Table S5. List of potential targets.**

| **No.** | **Uniprot ID** | **Gene** | **Degree** | **Betweenness Centrality** | **Closeness Centrality** |
| --- | --- | --- | --- | --- | --- |
| 1 | P31749 | AKT1 | 9 | 0.334 | 0.682 |
| 2 | P03372 | ESR1 | 9 | 0.253 | 0.652 |
| 3 | P23443 | RPS6KB1 | 6 | 0.078 | 0.577 |
| 4 | P42345 | MTOR | 5 | 0.027 | 0.556 |
| 5 | P19838 | NFKB1 | 5 | 0.104 | 0.577 |
| 6 | Q92731 | ESR2 | 5 | 0.021 | 0.536 |
| 7 | Q9Y618 | NCOR2 | 5 | 0.151 | 0.500 |
| 8 | P08069 | IGF1R | 4 | 0.257 | 0.500 |
| 9 | P01375 | TNF | 4 | 0.014 | 0.517 |

**Supplementary References**

1. Chang, Y.-S., Jin, H.-G., Lee, H., Lee, D.-S. & Woo, E.-R. Phytochemical Constituents of the Root Bark from *Morus alba* and Their Il-6 Inhibitory Activity. *Nat. Prod. Sci.* **25**, 268-274 (2019).

2. Takasugi, M. *et al.* The structure of phytoalexins produced in diseased mulberry. *Koen Yoshishu-Tennen Yuki Kagobutsu Toronkai* **22**, 275-282 (1979).

3. Nomura, T. & Fukai, T. Studies on the Constituents of the Cultivated Mulberry Tree IV. On the Reaction Mechanism of Photo-oxidative Cyclization of Morusin. *Heterocycles* **9**, 635 (1978).

4. Wang, L., Gong, T. & Chen, R. Y. Two new prenylflavonoids from *Morus nigra* L. *Chin. Chem. Lett.* **20**, 1469-1471 (2009).

5. Nomura, T., FUKAI, T. & KATAYANAGI, M. Kuwanon A, B, C and oxydihydromorusin, four new flavones from the root bark of the cultivated mulberry tree (*Morus alba* L.). *Chem. Pharm. Bull.* **25**, 529-532 (1977).

6. Zhang, Y. L., Luo, J. G., Wan, C. X., Zhou, Z. B. & Kong, L. Y. Four New Flavonoids with α‐Glucosidase Inhibitory Activities from *Morus alba* var. tatarica. *Chem. Biodivers.* **12**, 1768-1776 (2015).

7. Tran, H. N. K. *et al.* Anti-inflammatory activities of compounds from twigs of *Morus alba*. *Fitoterapia* **120**, 17-24 (2017).

8. Zheng, Z. P., Cheng, K. W., To, J. T. K., Li, H. & Wang, M. Isolation of tyrosinase inhibitors from *Artocarpus heterophyllus* and use of its extract as antibrowning agent. *Mol. Nutr. Food Res.* **52**, 1530-1538 (2008).

9. Sun, J. *et al.* Antioxidant and nitrite-scavenging capacities of phenolic compounds from sugarcane (*Saccharum officinarum* L.) tops. *Molecules* **19**, 13147-13160 (2014).

10. Chun-Nan, L. & Wen-Liang, S. Pyranoflavonoids from *Artocarpus communis*. *Phytochemistry* **31**, 2922-2924 (1992).

11. Lin, C.-N., Lu, C.-M., Lin, H.-C., Ko, F.-N. & Teng, C.-M. Novel antiplatelet naphthalene from *Rhamnus nakaharai*. *J. Nat. Prod.* **58**, 1934-1940 (1995).

12. Lee, S.-B. *et al.* Kaempferol 7-O-β-D-glucoside isolated from the leaves of *Cudrania tricuspidata* inhibits LPS-induced expression of pro-inflammatory mediators through inactivation of NF-κB, AP-1, and JAK-STAT in RAW 264.7 macrophages. *Chem.-Biol. Interact.* **284**, 101-111 (2018).

13. Lin, H.-Y., Kuo, Y.-H., Lin, Y.-L. & Chiang, W. Antioxidative effect and active components from leaves of Lotus (*Nelumbo nucifera*). *J. Agric. Food Chem.* **57**, 6623-6629 (2009).

14. Cao, X. *et al.* Isolation and purification of series bioactive components from *Hypericum perforatum* L. by counter-current chromatography. *J. Chromatogr. B* **879**, 480-488 (2011).

15. Pistelli, L., Spera, K., Flamini, G., Mele, S. & Morelli, I. Isoflavonoids and chalcones from *Anthyllis hermanniae*. *Phytochemistry* **42**, 1455-1458 (1996).

16. Yang, Z., Wang, Y., Wang, Y. & Zhang, Y. Bioassay-guided screening and isolation of α-glucosidase and tyrosinase inhibitors from leaves of *Morus alba*. *Food Chem.* **131**, 617-625 (2012).

17. Jung, J.-W. *et al.* Isoprenylated flavonoids from the root bark of *Morus alba* and their hepatoprotective and neuroprotective activities. *Arch. Pharm. Res.* **38**, 2066-2075 (2015).

18. Nguyen, N. H. *et al.* Isolation and characterization of three natural compounds from the stem bark of *Cassia grandis* Lf. *Can Tho University Journal of Science*, 57-60 (2016).

19. Jeong, S. H. *et al.* Tyrosinase inhibitory polyphenols from roots of *Morus lhou*. *J. Agric. Food Chem.* **57**, 1195-1203 (2009).

20. Li, H. X. *et al.* Identification of anti-melanogenesis constituents from *Morus alba* L. leaves. *Molecules* **23**, 2559 (2018).

21. Adfa, M., Yoshimura, T., Komura, K. & Koketsu, M. Antitermite activities of coumarin derivatives and scopoletin from *Protium javanicum* Burm. f. *J. Chem. Ecol.* **36**, 720-726 (2010).

22. Kuroyanagi, M. *et al.* Chemical studies on *Viburnum awabuki* K. KOCH. *Chem. Pharm. Bull.* **34**, 4012-4017 (1986).

23. Qin, B., Chen, Q.-P. & Lou, Z.-C. Active Constituents of *Viola prionantha* Bge. *J. Chin. Pharm. Sci.* **3**, 91 (1994).

24. Li, W., Koike, K., Asada, Y., Yoshikawa, T. & Nikaido, T. Biotransformation of umbelliferone by *Panax ginseng* root cultures. *Tetrahedron Lett.* **43**, 5633-5635 (2002).

25. Jin, W.-Y. *et al.* Antioxidant compounds from twig of *Morus alba*. *Nat. Prod. Sci.* **8**, 129-132 (2002).

26. Hakim, E. H. *et al.* Regioselective glucosylation of oxyresveratrol by cell suspension cultures of *Solanum mammosum*. *J Chem Res* **2004**, 706-707 (2004).

27. Kanchanapoom, T. *et al.* Stilbene and 2-arylbenzofuran glucosides from the rhizomes of *Schoenocaulon officinale*. *Chem. Pharm. Bull.* **50**, 863-865 (2002).

28. Su, B.-N. *et al.* Constituents of the Bark and Twigs of *Artocarpus dadah* with Cyclooxygenase Inhibitory Activity. *J. Nat. Prod.* **65**, 163-169 (2002).

29. Kim, H. *et al.* NMR analysis of lignins in CAD-deficient plants. Part 1. Incorporation of hydroxycinnamaldehydes and hydroxybenzaldehydes into lignins. *Org. Biomol. Chem.* **1**, 268-281 (2003).

30. Mendelson, W. L. & Hayden, S. Preparation of 2, 4-dihydroxybenzaldehyde by the Vilsmeier-Haack reaction. *Synth. Commun.* **26**, 603-610 (1996).

31. Li, L. & Seeram, N. P. Maple syrup phytochemicals include lignans, coumarins, a stilbene, and other previously unreported antioxidant phenolic compounds. *J. Agric. Food Chem.* **58**, 11673-11679 (2010).

32. Wu, Z.-J., Ouyang, M.-A. & Wang, S.-B. Two new phenolic water-soluble constituents from branch bark of *Davidia involucrata*. *Nat. Prod. Res.* **22**, 483-488 (2008).

33. Hiltunen, E., Pakkanen, T. T. & Alvila, L. Phenolic extractives from wood of birch (*Betula pendula*) (Walter de Gruyter, 2004).

34. Hisamoto, M., Kikuzaki, H. & Nakatani, N. Constituents of the leaves of *Peucedanum japonicum* Thunb. and their biological activity. *J. Agric. Food Chem.* **52**, 445-450 (2004).

35. Fujioka, S. *et al.* Isolation and identification of nicotinic acid as a flower-inducing factor in Lemna. *Plant Cell Physiol.* **27**, 103-108 (1986).

36. Abou-Hussein, D. R., Badr, J. M. & Youssef, D. T. Nucleoside constituents of the Egyptian tunicate *Eudistoma laysani*. *Nat. Prod. Sci.* **13**, 229-233 (2007).

37. Kim, S.-Y. *et al.* Effect of pinoresinol and vanillic acid isolated from *Catalpa bignonioides* on mouse myoblast proliferation via the Akt/mTOR signaling pathway. *Molecules* **27**, 5397 (2022).

38. Kim, S.-Y., Lee, J.-H., Kang, N., Kim, K.-N. & Jeon, Y.-J. The effects of marine algal polyphenols, phlorotannins, on skeletal muscle growth in C2C12 muscle cells via smad and IGF-1 signaling pathways. *Marine drugs* **19**, 266 (2021).

39. Oh, M., Kim, S.-Y., Park, S., Kim, K.-N. & Kim, S. H. Phytochemicals in Chinese chive (*Allium tuberosum*) induce the skeletal muscle cell proliferation via PI3K/Akt/mTOR and smad pathways in C2C12 Cells. *Int. J. Mol. Sci.* **22**, 2296 (2021).
